# Pervasive translational regulation of the cell signalling circuitry underlies mammalian development

**DOI:** 10.1038/ncomms14443

**Published:** 2017-02-14

**Authors:** Kotaro Fujii, Zhen Shi, Olena Zhulyn, Nicolas Denans, Maria Barna

**Affiliations:** 1 Department of Developmental Biology, Stanford University, Stanford, California 94305, USA; 2 Department of Genetics, Stanford University, Stanford, California 94305, USA

## Abstract

The degree and dynamics of translational control during mammalian development remain poorly understood. Here we monitored translation of the mammalian genome as cells become specified and organize into tissues *in vivo*. This identified unexpected and pervasive translational regulation of most of the core signalling circuitry including Shh, Wnt, Hippo, PI3K and MAPK pathways. We further identify and functionally characterize a complex landscape of upstream open reading frames (uORFs) across 5′-untranslated regions (UTRs) of key signalling components. Focusing on the Shh pathway, we demonstrate the importance of uORFs within the major SHH receptor, *Ptch1*, in control of cell signalling and neuronal differentiation. Finally, we show that the expression of hundreds of mRNAs underlying critical tissue-specific developmental processes is largely regulated at the translation but not transcript levels. Altogether, this work reveals a new layer of translational control to major signalling components and gene regulatory networks that diversifies gene expression spatially across developing tissues.

## Abstract (web summary)

Gene expression is regulated at several levels, including through the modulation of protein translation. Here the authors find that translation control diversifies gene expression between developing tissues and regulates major signalling pathways through a complex landscape of upstream open reading frames (uORFs).

A key question in developmental and stem cell biology is how information encoded in the genome is differentially expressed in time and space to give rise to diverse cell types and tissues. Over the last decades, numerous studies have demonstrated multiple layers of regulation, at both transcriptional and epigenetic levels, that govern when and where gene products are turned ‘on' and ‘off' to orchestrate specification and precise arrangement of distinct cell types into unique tissues and organs. By providing a highly tunable mechanism to control the abundance of final effector proteins after the birth of an mRNA, translational control may provide an additional layer of intricate regulation in tissue patterning and organogenesis^1^,^2^,^3^. However, both the magnitude and mechanisms for transcript-specific translation regulation remain poorly understood in the context of mammalian development. There is a lack of understanding for how translational control may drive key cell fate decisions, contribute to complex body plan formation, and underlie evolutionary diversity. Our current understanding of translational regulation is guided, to a large extent, by studies in cell culture or the earliest stages of development, before the initiation of zygotic transcription, where decisive events are solely directed by translation of pre-existing maternal mRNAs^4^. An example is the miRNA induced translational repression of maternal transcripts, which has been observed at the maternal-to-zygotic transition in early stages of zebrafish development^5^. In contrast, early mammalian development relies on zygotic transcription from the two-cell stage^6^, and hence the requirement for translational control during cell specification, organogenesis and pattern formation has been less certain.

In this work, we have carried out a comprehensive analysis of translational regulation of the mammalian genome within key lineages and tissues during mouse embryonic development. First, by zooming in on an important single lineage at mid-gestation, the mesoderm, we present evidence of prevalent translational regulation. One of the most significant networks of translationally regulated mRNAs unexpectedly belongs to cell signalling. We show that core signalling pathways, such as Shh, Wnt, Hippo, PI3K and FGF, which govern cell fate specification, proliferation and differentiation, are at the epicentre of translational control. Extending these findings, we functionally delineate broad translational repression of signalling circuits mediated, in part, through complex landscapes of upstream open reading frames. Further, by using the Shh signalling pathway as a paradigm example, we demonstrate that the stringent translational control imbued by uORFs within the 5′-UTR of the major SHH receptor *Ptch1* is critical for regulating Shh pathway activity and neuronal differentiation. Finally, we demonstrate that translational regulation within mammalian embryos represents a comprehensive regulatory cascade that further diversifies gene expression spatially across tissues within the same stage of embryonic development. In particular, by further carrying out ribosome profiling within distinct tissues such as the neural tube as well as the developing limb bud our studies show that hundreds of mRNAs guiding critical tissue-specific functions are regulated largely at the translation but not transcript level. Importantly, a large number of translationally regulated mRNAs guide key tissue-specific developmental processes. Altogether, these studies reveal a new layer of translational control to major signalling networks and key developmental patterning genes that diversifies the expression of a relatively fixed number of genes that control tissue patterning and development.

## Results

### Translational regulation of the cell signalling circuitry

**Figure 1:**
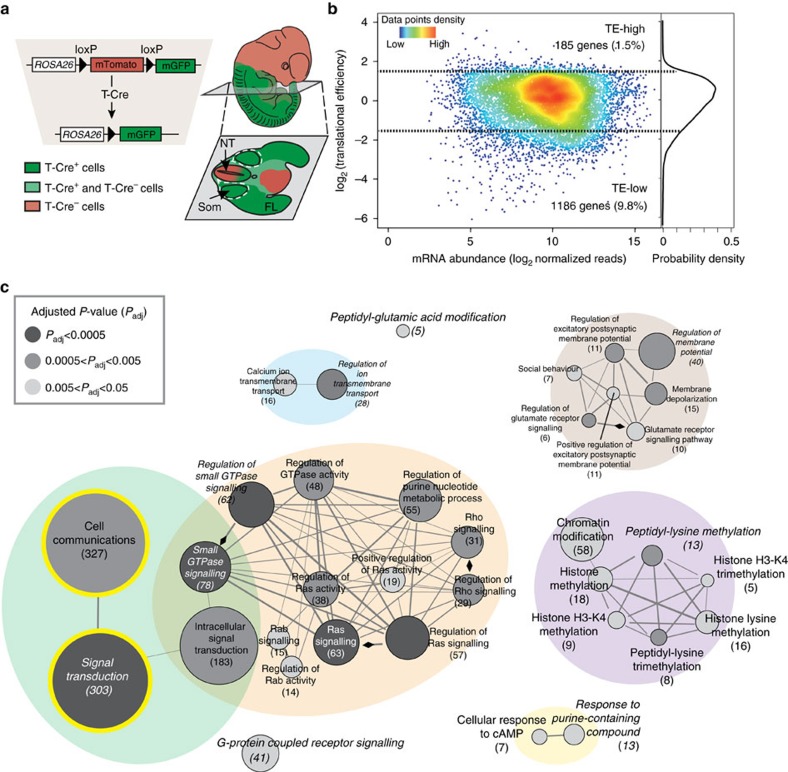
Ribo-Seq in parallel with RNA-Seq reveals extensive translational regulation of key signalling components. (**a**) Double-fluorescent T-Cre reporter system allows marking of mesodermal lineage. T-Cre mediates the excision of the ubiquitously expressed mTomato cassette which results in expression of mGFP. Cross section shows distribution of GFP^+^ mesodermal cells in the E11.5 embryo. Arrows and labels indicate NT (neural tube), Som (somites), and FL (forelimbs). (**b**) The distribution of log_2_TE (translational efficiency) over mRNA abundance (log_2_ normalized reads) in the mesodermal lineage at E11.5, with the densities of data points indicated as colours. 1,186 and 185 genes comprising 9.8 and 1.5% of the total analysed genes are defined as TE-low and TE-high respectively, whose TE is significantly lower or higher than the median (false-discovery rate (FDR)<0.05) and the difference at least threefolds. (**c**) The network of enriched GO categories (biological process) among TE-low genes clusters into several functional groups represented by circles of different colours. Each node represents one enriched GO category colour-coded by its adjusted *P*-value (FDR) of enrichment, with the size of the node proportional to the number of associated TE-low genes. Edges indicate similarity (Kappa score >0.4) between the two connected GO categories. Edges with an arrowhead indicate a regulation relationship between nodes. The number of TE-low genes is shown in the parenthesis underneath the name of each GO term. The most significantly enriched GO category from each group is designated as the leading group term and highlighted in italic. Signal transduction and cell communication categories are highlighted with a yellow outline.

To simultaneously quantify the abundance of total mRNAs and ribosome-bound mRNAs undergoing translation as cells become specified and organize into distinct organs in mammalian embryos at a genome-wide level, we conducted RNA sequencing (RNA-Seq) in parallel with ribosome profiling (Ribo-Seq)^7^. At first, we examined the transcription and translation profiles of the mesoderm, one of the three germ layers of the mammalian embryo. The mesoderm gives rise to wide array of cell and tissue types, including muscle, cartilage and bone, urogenital structures, connective tissue, as well as heart and blood cells. We used the double-fluorescent T-Cre (T-Cre; mT/mG) reporter system in which membrane-bound Tomato (mT) is expressed in all cells of the mouse embryo before Cre-activation and membrane-targeted enhanced green fluorescent protein (mG) is expressed after activation^8^ of T-Cre, which labels the mesodermal lineage derived from the primitive streak^9^. This enabled us to mark all of the lineages derived from the paraxial mesoderm (somites), lateral plate mesoderm (limbs) and intermediate mesoderm (nephrons), and to isolate the GFP^+^ cells by fluorescence activated cell sorting (FACS; Fig. 1a; Supplementary Fig. 1a,b). For both RNA-Seq and Ribo-Seq, we performed a total of three biological replicates (Supplementary Data 1), and obtained highly consistent data between replicates with pairwise Pearson's correlation between 0.91 and 0.99 (Supplementary Fig. 2a,b). We find that our Ribo-Seq analysis encompasses reads that have a discrete size (∼30 nt - the size of ribosome footprint), a 3-nt periodicity and mainly mapped to the coding DNA sequence (CDS) (∼80%), all of which show that our Ribo-Seq data set is of good quality to study translational control *in vivo* (Supplementary Fig. 3a–c)^7^,^10^. Metagene analysis of read distribution around the beginning and end of the CDS also indicated a pileup of ribosome-protected fragments (RPFs) at the beginning of the CDS (Supplementary Fig. 3d), plausibly caused by the cycloheximide treatment. Therefore, we excluded the first 15 or last 5 codons of each transcript to ensure analysis of the coding regions that is most reliable for differential expression analysis similar to previous publications^10^,^11^.

We focused on translational control of gene expression in the mesoderm at E11.5 when the cells of this lineage undergo major specification and differentiation events directed by a myriad of signalling cues, including FGFs, Wnts, and Shh, as they fully egress from the primitive streak, migrate, and differentiate along the anteroposterior (A–P) axis of the developing embryo. Examining the expression of lineage-specific markers ensured the quality of isolation of the desired mesodermal cell population (Supplementary Fig. 4). To obtain a global view of gene regulation at the translational level, we calculated translational efficiency (TE) by comparing the level of RPFs with mRNA abundance on the CDS of each gene (Fig. 1b). In brief, applying the framework of the generalized linear model (GLM) in the DESeq statistical package for analysing sequencing count data^12^,^13^, a linear regression was performed to the normalized read counts, as a function of library type variables (‘RNA-Seq' or ‘Ribo-Seq'). Here the coefficient of library type variables (‘Ribo-Seq' over ‘RNA-Seq') is a measurement of TE (see Methods). This revealed a wide distribution in the TE, with over a 10-fold difference between the 5th percentile of most actively compared with the 5th percentile of the least actively translated genes, suggesting extensive regulation at the step of mRNA translation in the mesoderm lineage of developing mammalian embryos. Specifically, we identified 1,186 and 185 genes comprising 9.8 and 1.5% of the total analysed genes whose TE is significantly lower or higher than the median (false-discovery rate (FDR)<0.05) and the difference is at least threefold. (Fig. 1b; Supplementary Data 2), designated as TE-low and TE-high gene sets, respectively.

**Figure 2:**
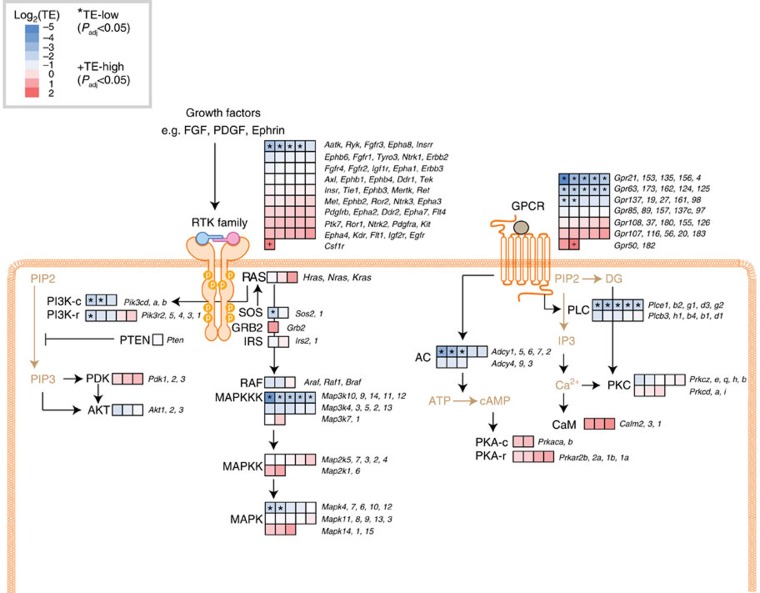
The intracellular signalling network in response to growth factors as a paradigm of translational control. Shown is an integrative view of all core signalling components of the growth factor signalling pathway. Each square represents one gene paralogue of the indicated gene family (except for the RTK (receptor tyrosine kinase) which contains multiple gene families, where each square represents one gene encoding an RTK). The translational efficiency of each gene in the mesoderm at E11.5 is shown by colour-coding in a blue (TE-low) to red (TE-high) scale. PI3K-c/PKA-c and PI3K-r/PKA-r refer to their catalytic and regulatory subunits respectively. The *or^+^mark genes that have significantly low (TE-low) or high (TE-high) translational efficiency as defined in Fig. 1. Pointed arrows or blunt arrows indicate either stimulation or inhibition in signal transduction.

To understand biological processes subject to this extensive degree of translational control, we performed gene ontology (GO) analysis of genes that are TE-low and TE-high in the mesoderm. This revealed many significantly enriched GO categories (FDR<0.05; Fig. 1c; Supplementary Data 3 and 4), which were further grouped into several major functional clusters most represented in TE-low genes by: signal transduction (green), regulation of small GTPase mediated signal transduction (orange), regulation of membrane potential (brown), regulation of ion transmembrane transport (blue), peptidyl-lysine methylation (purple), response to purine-containing compound (yellow) and G-protein coupled receptor signalling pathway (Fig. 1c). Over 40% of all TE-low genes belong to at least one of the enriched GO categories, indicating the coordinated translational regulation of genes with related functions (Supplementary Data 4). Notably, ‘signal transduction', comprised of 303 genes (a child term of the ‘cell communication' GO term comprised of 327 genes), is one of the largest and most significantly enriched functional categories of all TE-low genes. A molecular function GO enrichment analysis of the TE-low genes further underscores that key enzymes involved in cell signalling cascades (GTPases, kinases and many others) are the main targets of translational repression (Supplementary Fig. 5 and Supplementary Data 5). The top enriched categories include protein kinase activity, GTPase regulator activity, phospholipid binding, signalling receptor activity, cytoskeleton binding, gated channel activity, and Ras GTPase activity. One striking example of a translationally regulated cell signalling pathway is the receptor tyrosine kinase (RTK) network initiated upon binding and activation by specific growth factors (for example, FGF, PDGF, Ephrin), which then elicits signalling from the RAS-RAF-MEK-ERK and phosphatidylinositide 3-kinase (PI3K) arms of the network. Important insight into the potential impact of translational regulation within this cell signalling pathway emerges from the analysis of the individual translational efficiency of each mRNA within this network (Fig. 2 and Supplementary Data 6). First, most enzymes required for the production of major classes of secondary messengers are found to be translationally regulated. In contrast, none of the direct effector proteins responding to these secondary messengers show significant regulation at the translational level. For instance, multiple isoforms of PI3K, which are required for catalysing the synthesis of phosphatidylinositol (3,4,5)-trisphosphate (PIP3) from phosphatidylinositol 4,5-bisphosphate (PIP2), have significantly lower TE (Fig. 2; *indicates TE-low, FDR<0.05). In contrast, downstream effectors, such as PDK or AKT, that respond to PIP3 do not. These findings suggest that these upstream components of cell signalling may be subject to more stringent translational control than downstream components. Supporting this hypothesis, in the G-protein coupled receptor (GPCR) signalling cascade, 12/32 receptors, 5/10 phospholipase C (PLC) enzymes and 3/8 adenyl cyclases (AC) are TE-low, conversely none of the more downstream second messenger responding components exhibit significant changes in TE (Fig. 2). Since upstream components are often the most rate limiting and dose-sensitive within signalling cascades, their regulation at the level of translation may reflect a molecular programme that promotes rapid and efficient control of the overall output of the pathway. Secondly, our analysis revealed a wide range in translational efficiencies among homologues within specific families of signalling proteins. For example, the GPCR and MAPKKK homologues exhibit ∼80- and ∼20-fold difference in TE (Fig. 2). Given the large number of gene homologues belonging to these gene families, translational control may provide greater specificity in the strength or activity of selective groups of these receptors and kinases over others in regulation of cell signalling.

**Figure 3:**
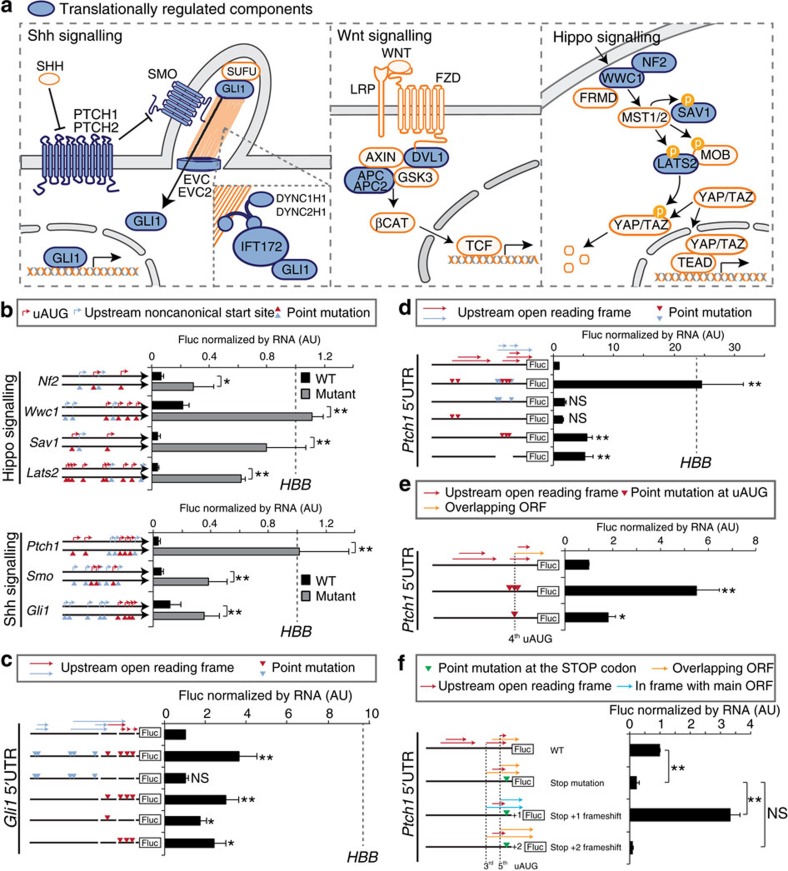
Characterization of uORFs as potent *cis*-acting repressors in the translational regulation of signalling pathway genes. (**a**) Multiple core components of Shh, Wnt and Hippo pathways that are under significant translational regulation are shown in blue. Pointed and blunt arrows indicate stimulation or inhibition. (**b**) Firefly luciferase (Fluc) reporter activity downstream of 5′-UTRs of Hippo or Shh pathway genes. Values are shown relative to *HBB* 5′-UTR construct indicated by the dashed line. Full-length wildtype 5′-UTRs contain multiple uAUGs (red arrows) and non-canonical initiation sites within a strong Kozak context (blue arrows). Black bars indicate activity of wildtype 5′-UTR, grey bars indicate activity of mutated 5′-UTRs in which point mutations were generated in all uAUGs (red triangles) and non-canonical initiation sites (blue triangles). (**c**–**f**) Values are shown relative to wildtype 5′-UTR constructs: uORFs initiating at uAUG (red arrows); uORFs initiating from non-canonical start sites (blue arrows); point mutations in uAUGs (red triangle); point mutations in non-canonical start sites (blue triangles). (**c**) Fluc activity downstream of wildtype and mutant *Gli1* 5′-UTR (**d**) or *Ptch1* 5′-UTR. (**e**) uORF overlapping with main ORF (oORF) is indicated by yellow arrows. Point mutation in the 4th uAUG reveals that it is not important for translational repression of *Ptch1* 5′-UTR. (**f**) uORFs that are in frame with main ORF are indicated by blue arrows; oORF (yellow arrows). A green triangle indicates the location of a point mutation (tg‘T'GA to tg‘A'GA). This point mutation disrupts the upstream stop codon for the 3rd and 5th uAUGs changing the two corresponding uORFs to oORFs and producing a new stop codon following the 4th uAUG changing its corresponding sequence from an oORF to an uORF. The insertion of 1 nt (‘Stop+1 frameshift') just before the CDS shifts the 3rd and 5th uAUGs in frame with the main ORF. The insertion of 2 nt (‘Stop+2 frameshift') just before the CDS shifts these uAUGs back out of frame. For data in panels (**b**–**f**) assays were performed in NIH3T3 cells. Fluc reporter activity was normalized to Fluc mRNA and transfection efficiency using co-transfected Renilla luciferase (Rluc) normalized to Rluc mRNA. Error bars represent s.d. AU, arbitrary unit; **P<0.01; *P<0.05; NS, not significant (*t*-test, *n*≥4).

An additional, unexpected finding from our ribosome profiling analysis is that most of the core toolbox of developmental cell signalling pathways, which are reutilized at multiple times and places to control fundamental aspects of cell specification and tissue patterning, including Shh, Wnt, Hippo and FGF, contained multiple core components under translational control (Figs 2 and 3a). This was best represented by *Ptch1*, *Ptch2*, *Smo*, *Gli1* and components of the primary cilia in the Shh pathway, *Dvl1*, *Apc*, *Apc2* and *Rspo1* in the Wnt pathway, as well as *Nf2*, *Wwc1*, *Sav1*, *Lats2,* and *Wtip* in the Hippo pathway (Fig. 3a). Despite numerous lines of research into these important developmental signalling cascades, the main focus has been their regulation at the transcriptional and post-translational levels. These findings suggest that translational control of key developmental signalling pathways may reflect a new point of regulation guiding mammalian development.

### A complex landscape of uAUGs within signalling components

To study the impact of translational control on cell signalling as well as cell fate determination, we chose the Shh and Hippo pathways (RNA-Seq and Ribo-Seq tracks are shown in Supplementary Fig. 6) as examples to first understand how transcript-specific translational regulation is achieved at the molecular level. Notably, TE-low transcripts overall had substantially longer 5′-UTRs but not 3′-UTRs, and contained a significantly higher number of upstream AUGs (uAUGs) in their 5′-UTRs, compared with all transcripts analysed (Supplementary Fig. 7). We therefore focused on the 5′-UTRs of many core components of key signalling pathways. As many of these signalling genes contain long and poorly characterized 5′-UTRs, which could harbour sequences that enable translational control, we performed 5′ RACE (rapid amplification of cDNA ends) on embryonic tissues and cloned the full-length 5′-UTR sequences of multiple components of the Shh and Hippo signalling pathways, focusing on the predominant transcript isoforms expressed in the mesoderm lineage (Supplementary Fig. 8). We observed that the 5′-UTR sequences of translationally repressed components of the Hippo (*Nf2* (551 nt), *Wwc1* (512 nt), *Sav1* (371 nt) and *Lats2* (436 nt)) and Shh (*Ptch1* (841 nt), *Smo* (514 nt) and *Gli1* (665 nt)) pathways repressed translation of Firefly luciferase (Fluc) relative to the control 5′-UTR of *HBB* (human haemoglobin beta; Fig. 3b), extending our Ribo-Seq results and suggesting that translational repression is encoded, at least in part, in their 5′-UTRs. Close examination of these 5′-UTRs revealed the presence of multiple uAUGs (Fig. 3b; Supplementary Table 1). To mechanistically assess the translational regulation mediated by uAUGs, we systematically mutated all uAUGs, as well as non-canonical start sites having a favourable Kozak sequence, since these codons also have the potential to contribute to regulation^14^, and compared their translational efficiency to wildtype 5′-UTRs. We observed a marked increase in Fluc translation across all assayed components of the Hippo (*Nf2*, *Wwc1*, *Sav1* and *Lats2*) and Shh pathways (*Ptch1*, *Smo* and *Gli1*; Fig. 3b) revealing that multiple components of the core signalling machinery of the cell are under stringent translational control, which is mediated in part by a complex landscape of upstream initiation sites.

### uORF repression of *Ptch1* controls neural differentiation

Although recent studies have identified putative uORFs genome-wide^15^,^16^,^17^, little is known about the functional contribution of uORF-mediated regulation, particularly in the context of mammalian development. By far, the best example of uORF-mediated repression with biological significance remains that of regulation of yeast GCN4 mRNA in response to stress^17^,^18^. To further understand how upstream initiation sites contribute to translational repression, we selected *Ptch1* and *Gli1* transcripts as examples for further analysis. *Ptch1* encodes the main receptor of the SHH ligand and acts as strong negative regulator of the Shh signalling pathway. Conversely, *Gli1* is a transcription factor, which acts as a downstream activator of the pathway. The expression of both genes is tightly controlled at both transcriptional and post-translational levels and has been well studied both *in vitro* and *in vivo* making them excellent examples to study the role of translational control in the context of complex gene regulatory landscapes. The following trends emerged from our analysis, first, uAUGs of the *Ptch1* 5′-UTR are overall highly conserved, in comparison with non-canonical start codons (Supplementary Fig. 9). Indeed, mutations of non-canonical start codons showed that they have little impact on translation of the main ORF (Fig. 3c,d). Second, uAUGs occur in clusters in both the 5′-UTR of *Gli1* and *Ptch1* (Fig. 3c,d). With respect to *Ptch1*, deletion of the first uAUG cluster had no effect on translation (Fig. 3d). In contrast, deletion of the second cluster resulted in approximately a sixfold increase in translational efficiency (Fig. 3d) with low contribution from the 4th uAUG (Fig. 3e). Finally, an upstream stop codon mutagenesis, which placed the uAUGs in frame with the main ORF, allowed us to precisely delineate that the 3rd and/or 5th uAUGs are capable of initiating translation. These findings define repressive uORFs in the *Ptch1* 5′-UTR that act to downregulate translation from the main AUG by competing away translationally competent ribosomes (Fig. 3f).

**Figure 4:**
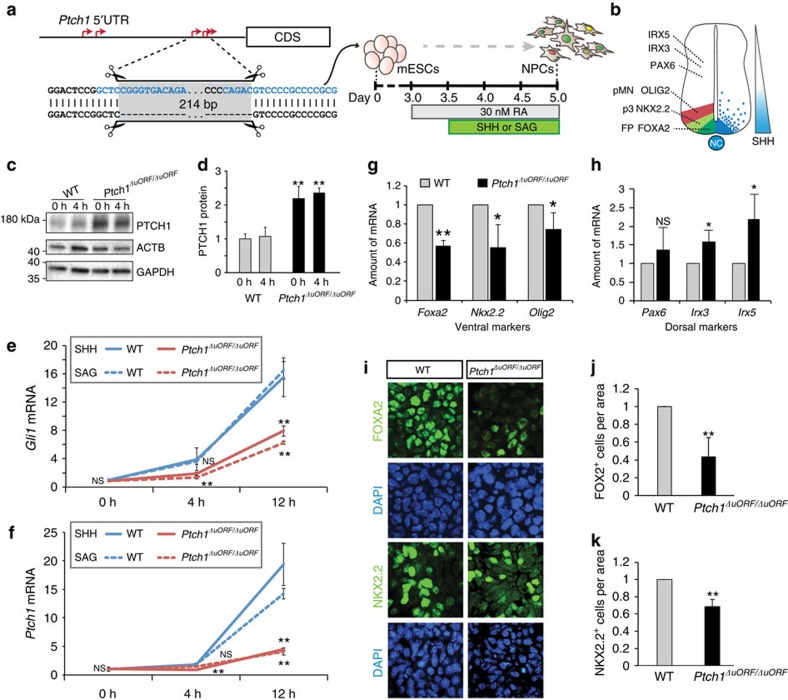
Loss of uORF-mediated translational regulation of *Ptch1* disrupts Shh signalling and neurogenesis. (**a**) The position guide RNA (gRNA) targets for the generation of *Ptch1*^*ΔuORF/ΔuORF*^ homozygous mutant mESC line with CRISPR/Cas9 is shown on the schematic accompanied by the sequence flanking the deletion. gRNA target sequence is indicated in blue. Schematic of monolayer differentiation of mESCs into neural progenitor cells (NPCs) is depicted. RA (Retinoic Acid) treatment is initiated on day 3.0. Shh pathway activation to generate ventral neurons is achieved by stimulation with either recombinant SHH protein or SAG beginning on day 3.5 of differentiation—assays in **c**–**f** are performed at day 3.5+0, 4, 12 h. Differentiation is terminated at day 5 (36 h) (**g**–**k**). (**b**) Schematic illustrates graded Shh signal across the ventral-to-dorsal axis of the developing neural tube. Highest concentration of SHH specifies FOXA2^+^ FP, progressively lower quantities of SHH specify NKX2.2^+^ p3 and OLIG2^+^ pMN neurons. PAX6^+^, IRX3^+^ and IRX5^+^ neurons form in the dorsal portion of the neural tube and are not SHH dependent. (**c**–**d**) Western blot analysis reveals increased PTCH1 protein at 0 and 4 h after SAG treatment. Analysis of (**e**) *Gli1* and (**f**) *Ptch1* mRNA expression at 0, 4 and 12 h after addition of SHH protein (solid lines) or SAG (dashed lines) on day 3.5 of NPC differentiation. (**g**) Analysis of mRNA expression of ventral neuron marker genes (*Foxa2*, *Nkx2.2* and *Olig2*) and (**h**) dorsal neuron maker genes (*Pax6*, *Irx3* and *Irx5*) by RT-qPCR in wildtype and *Ptch1*^*ΔuORF/ΔuORF*^ cells at day 5 of neuron differentiation. (**i**–**k**) Concomitant immunofluorescence staining and quantitation confirms decreased numbers of FOXA2^+^ and NKX2.2^+^ neurons at day 5 of differentiation. (**c**–**k**) Error bars represent s.d. (*t*-test, **P<0.01; *P<0.05; NS, not significant, *n*≥3).

In the developing embryo, the 5′-UTRs of *Ptch1* and *Gli1* act in the context of a tightly regulated signalling cascade characterized by well-defined feedback mechanisms. To understand the biological impact of uORF-mediated translational control, we used CRISPR-Cas9-mediated mutagenesis to generate a homozygous deletion encompassing the entire inhibitory uORF cluster in the 5′-UTR of *Ptch1* in mouse embryonic stem cells (mESCs; Fig. 4a). Since mESCs do not engage in active Shh signalling and express very little detectable *Gli1* and *Ptch1* transcript, we differentiated the *Ptch1* homozygous uORF deletion mutant (*Ptch1*^*ΔuORF/ΔuORF*^) mESCs precisely into ventral neurons. Unlike mESCs, ventral neurons express Shh pathway components and require exposure to very high levels of Shh signalling activation for their specification. As a consequence, ventral neurons are acutely sensitive to the level of pathway activation *in vitro*, mirroring the Shh-dependent ventral neuron differentiation evident within the developing neural tube, where PTCH1 acts as a strong negative regulator of the Shh signalling pathway (Fig. 4a,b)^19^,^20^,^21^. To obtain Shh-responsive ventral neurons we carried out a 5-day differentiation protocol whereby we first differentiated mESCs into Shh-sensitive neuronal precursor cells (NPCs; days 0–3 of differentiation). From day 3.5 to day 5 of differentiation, we treated NPCs with a high concentration of either recombinant SHH protein or SAG (Smoothened Agonist) to achieve high levels of pathway activity necessary for their differentiation into Shh-dependent ventral neuron lineages (Fig. 4a,b). To examine the extent to which PTCH1 protein levels change upon deletion of the uORF cluster, we differentiated wildtype and *Ptch1*^*ΔuORF/ΔuORF*^ mESCs into NPCs. At day 3.5 of differentiation we treated the cells with SAG, a small molecule activator of the Shh pathway, and carried out western blot analysis at 0 and 4 h after pathway activation. We observed that the level of PTCH1 protein was substantially elevated in *Ptch1*^*ΔuORF/ΔuORF*^ NPCs compared with wildtype controls already at both 0 and 4 h (Fig. 4c,d) indicating that the homozygous uORF deletion has a significant effect on PTCH1 protein levels in the native context. To assess how elevated PTCH1 protein levels affect Shh signalling, we examined expression of *Gli1* and *Ptch1* mRNA by quantitative PCR with reverse transcription (RT-qPCR) after 0, 4, and 12 h of treatment with either SAG or recombinant SHH protein (Fig. 4e,f). Since both *Gli1* and *Ptch1* are direct transcriptional targets of Shh signalling, their expression is typically used as a primary means of assessing pathway activation. We noted that treatment with either SHH or SAG resulted in increased expression of *Gli1* as early as 4 h (Fig. 4e) and *Ptch1* at 12 h in wildtype NPCs (Fig. 4f). In contrast, *Ptch1*^*ΔuORF/ΔuORF*^ NPCs exhibited markedly diminished *Gli1* mRNA transcription as early as 4 h compared with WT controls (Fig. 4e). Similarly,the expression of *Ptch1* mRNA at 12 h was lower in *Ptch1*^*ΔuORF/ΔuORF*^ NPCs compared with controls (Fig. 4f) consistent with diminished pathway activity in these cells. Notably *Ptch1*^*ΔuORF/ΔuORF*^ NPCs responded similarly to both SHH protein, which binds directly to PTCH1, and SAG, which binds to Smoothened (SMO). Since the role of PTCH1 is to inhibit SMO activation in absence of SHH, it is likely that the decreased responsiveness to Shh pathway stimulation observed in *Ptch1*^*ΔuORF/ΔuORF*^ NPCs is due to increased repression of SMO by elevated PTCH1 levels at steady state.

To investigate whether the development and differentiation of Shh-dependent neurons is affected as a result of the *Ptch1*^*ΔuORF/ΔuORF*^ mutation, we examined the expression of markers of ventral neuronal identity by RT-qPCR. NPC differentiation mirrors the signalling environment observed in the developing neural tube where exposure to the highest concentration of SHH ligand specifies the FOXA2^+^ floor plate (FP), while slightly lower concentrations give rise to the NKX2.2^+^ p3 interneurons and OLIG2^+^ pMN motor neuron progenitors (Fig. 4b)^19^,^20^. Consistent with our observation of decreased Shh signalling (Fig. 4e,f), we observed a concomitant decrease in the expression of Shh-dependent *Foxa2*, *Nkx2.2* and *Olig2* mRNAs (Fig. 4g) and a concomitant increase in Shh-independent dorsal neural tube markers such as *Irx3* and *Irx5* (Fig. 4h). Furthermore, these changes were associated with a decrease in the specification of FOXA2^+^ and NKX2.2^+^ neurons as assessed by immunostaining on day 5 of differentiation (Fig. 4i–k). Altogether, these studies functionally reveal the mechanisms guiding translational regulation of core components of developmental signalling pathways and their importance in control of cell signalling and the precise differentiation of key cell types.

### Translation control diversifies gene expression in tissues

**Figure 5:**
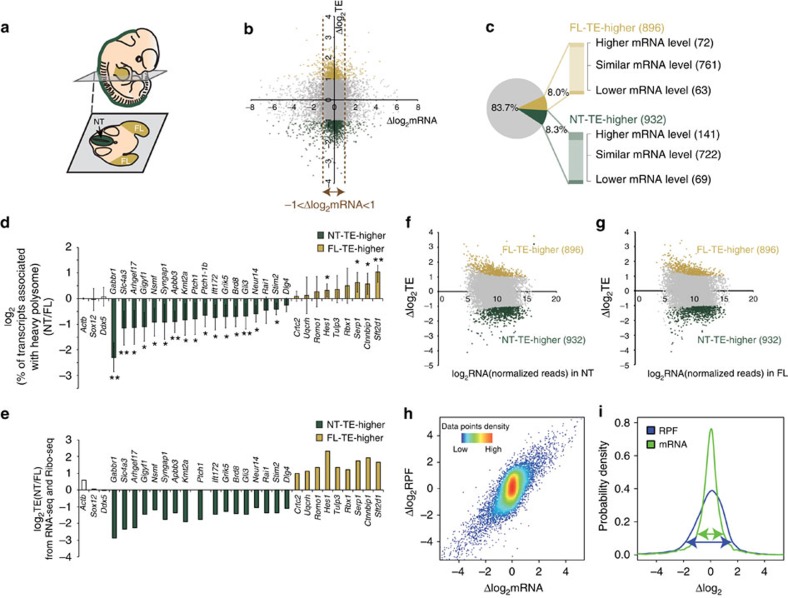
Snapshots of translational landscapes of developing mammalian embryos reveal dynamic translational control of signalling between tissues. (**a**) Schematic illustration of an E11.5 embryo. The neural tube (NT), in dark green, and forelimb (FL), in yellow, were microdissected and assessed for differences in translational efficiency (TE) at a genome-wide level by Ribo-Seq and RNA-Seq. (**b**) Scatter plot of Δlog_2_ in TE over Δlog_2_ in mRNA between the NT and FL. NT-TE-higher and FL-TE-higher are genes with significantly higher TE in NT (FDR<0.2 and Δlog_2_TE<−1) or higher TE in FL (FDR<0.2 and Δlog_2_TE>1), respectively. The vertical dash lines mark the region with no significant change in the mRNA levels in between (−1<Δlog_2_ mRNA<1). (**c**) Pie chart shows the number and percentage of genes having higher TE in the FL (yellow), higher TE in the NT (dark green) and no significant difference in TE (grey). Genes with distinct TEs are further divided according to the changes in the mRNA levels, shown in the column chart. (**d**) log_2_ change in the percentage of mRNAs associated with actively translating polysomes determined by quantifying mRNA in the heavy polysome fractions and normalized by its total. *Ptch1-1b* represents the predominant isoform of *Ptch1* in both in NT and FL (Supplementary Fig. 8), identified by 5′RACE. Error bars represent s.d. (*t*-test, **P<0.01; *P<0.05, *n*≥5). (**e**) Δlog_2_TE between NT and FL from Ribo-Seq and RNA-Seq analysis. (**f**,**g**) Scatter plot of Δlog_2_TE between tissues over the mRNA abundance (log_2_mRNA(normalized reads)) in (**f**) NT and (**g**) FL. (**h**) Shown is the distribution of Δlog_2_ in RPFs over the Δlog_2_ mRNA between NT and FL at E11.5. (**i**) Comparison of the probability density distribution of Δlog_2_ in mRNA (green) and RPFs (blue) reveals a significantly greater change in the RPFs than mRNAs between the tissues. (**b**–**i**) Colour key: higher TE in the FL (yellow), higher TE in the NT (dark green) and no significant difference in TE (grey).

Finally, we investigated the dynamics of spatial regulation of translational control between developing tissues within the mammalian embryo. The core developmental toolbox of cell signalling, for example, Wnt, Shh, FGF, BMP and Notch pathways, are reused in multiple tissues and at multiple times during development. The finding that a specific repertoire of core signalling components are significantly low in TE in the mesoderm lineage is intriguing and led us to ask whether the translational regulation could be distinct in specific cell/tissue types. To this end, we extended our Ribo-Seq analysis to assess translational control in additional developing tissues and in particular the neural tube and limb bud, which both contain major signalling centers. We performed RNA-Seq and Ribo-Seq on three biological replicates of precisely microdissected neural tubes (NT) and forelimb buds (FL) of mouse E11.5 embryos (Fig. 5a). All three biological replicates are highly consistent with pairwise Pearson's correlation between 0.89 and 0.99 (Supplementary Fig. 2a,b) and Ribo-Seq libraries exhibit typical characteristic features that ensure good quality of the data set for this analysis (Supplementary Fig. 3a–d). To reveal the differences in translational regulation between tissues, we analysed the interaction term of the library types (‘Ribo-Seq' and ‘RNA-Seq') with sample types (‘Neural tube' or ‘Forelimb') in the framework of the GLM model using DESeq. The coefficient of this interaction term is a measurement of changes in TE between tissues. This analysis identified significant differences in translation regulation between these tissues at the same developmental stage. Specifically, 932 (8.3%) of genes displayed a significantly higher TE in the NT compared with FL and 896 (8.0%) of genes displayed a significantly higher TE in the FL compare with the NT, respectively, with a fold-change ≥2 and FDR<0.2 (Fig. 5b,c; Supplementary Data 7). Importantly, we observed that NT-TE-higher transcripts indeed have higher association with actively translating heavy polysomes in NT, whereas FL-TE-higher transcripts were more associated with heavy polysomes in FL (Fig. 5d,e; Supplementary Fig. 10), further corroborating the Ribo-Seq and RNA-Seq analysis. Interestingly, the vast majority (∼80%) of genes with distinct TE between tissues exhibit mild difference in mRNA levels (fold-change <2; Fig. 5b,c), indicating that translational control predominantly regulates the expression of this set of genes. Furthermore, both the NT-TE-higher and FL-TE-higher transcripts exhibit a wide distribution of their mRNA abundance in both tissues (Fig. 5f,g), with comparable median gene expression levels compared with all other transcripts, and are not simply lowly expressed transcripts that could be more easily confounded by higher variances in their expression. This suggests that translational regulation, in addition to simply buffering differences from transcription as observed in previous studies^22^,^23^, represents an important mechanism to further diversify gene expression profiles between different tissues in the developing mammalian embryo (Fig. 5h,i).

**Figure 6:**
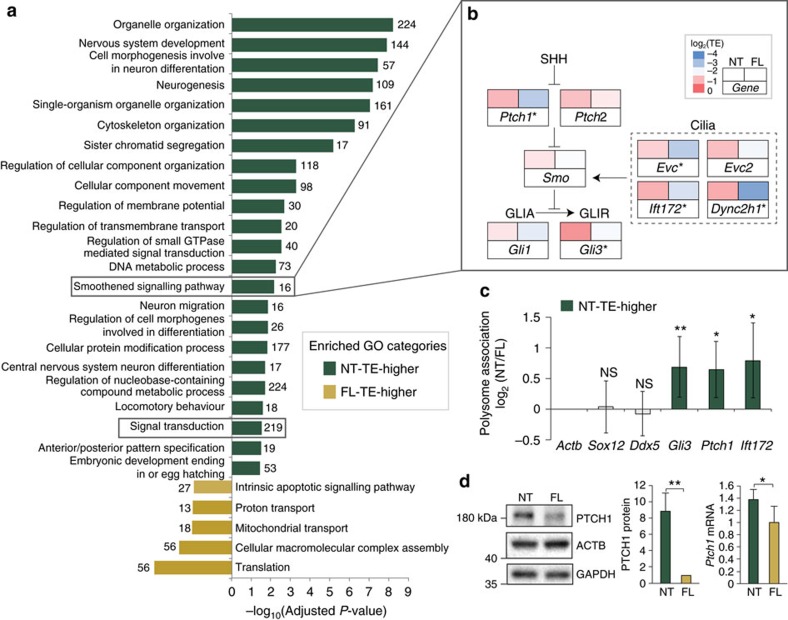
Translational regulation of the Shh signalling pathway between tissues. (**a**) Bar graph showing the enriched GO categories of genes with higher TE in the neural tube (NT) in dark green and forelimb (FL) in yellow. The height of each bar shows the significance of enrichment and the number of associated genes in each GO categories with either higher TE in the NT or FL is indicated. Grey boxes highlight the ‘Smoothened signalling pathway' and ‘Signal transduction' GO categories. (**b**) Comparison of translational efficiency (TE) of Shh pathway components between tissues. Adjacent boxes depict neural tube (NT) and forelimb (FL) TE values colour-coded as shown in the legend. The *marks TE-changed genes, as defined in Fig. 5 (|Δlog_2_TE|>1 and FDR<0.2). (**c**) Translation efficiency analysis by orthogonal assay shows increased polysome association of *Ptch1*, *Gli3* and *Ift172* transcripts in NT compared with FL lysates confirming increased TE for these transcripts in the NT. (**d**) Western blot and RT-qPCR analysis confirms that PTCH1 protein levels are increased in the NT relative to the FL with mild change in *Ptch1* mRNA levels. (**c**,**d**) Error bars represent s.d. (*t*-test, **P<0.01; *P<0.05, *n*≥5).

To further understand the translational control programme unique to different tissue types, we performed GO enrichment analysis to delineate the general functional categories of genes with significantly higher TE in either the NT or the FL (NT-TE-higher and FL-TE-higher), respectively (Fig. 6a; Supplementary Data 8). Interestingly, over 200 NT-TE-higher genes belong to the ‘signal transduction' and ‘Smoothened signalling pathway' GO categories, including multiple core components of the Shh pathway: *Ptch1*, *Gli3*, *Ift172*, *Evc* and *Dync2h1* (Fig. 6b). Consistent with this, we observe significantly higher association of these transcripts with translating polysomes in NT compared with FL (Fig. 6c). Direct examination of the levels of PTCH1 in NT and FL reveals a substantially higher level of PTCH1 protein in the developing neural tube despite comparable levels of *Ptch1* transcript (Fig. 6d). The difference in translational regulation of signal transduction genes suggests that a specific reservoir of mRNAs translationally repressed in one tissue type may become de-repressed in another. This may reflect a highly tunable programme of translational control directing unique tissue-specific programs of cell signalling and development. In support of this hypothesis, among the enriched GO categories of NT-TE-higher genes, we observe functional categories belonging to ‘nervous system development', ‘regulation of membrane potential', ‘neurogenesis', ‘neuron migration and differentiation', ‘central nervous system neuron differentiation' and ‘locomotory behaviour' the majority of which have similar mRNA levels between the limb bud and neural tube (Fig. 5b,c). This enrichment of neuron-related functions among the NT-TE-higher genes highlights the unexpected translational control of mRNAs that are designated with tissue-specific functions. However, other GO categories of NT-TE-higher genes such as ‘Sister chromatid exchange' and ‘DNA metabolic process' suggest a more complex picture of translationally regulated mRNAs in the neural tube that is not obviously related to known neural specific functions. Moreover, although there are hundreds of translationally regulated mRNAs in the FL, relatively fewer enriched GO categories of FL-TE-higher genes emerge. Interestingly, however, functional categories associated with FL-TE-higher genes include the ‘intrinsic apoptotic signalling pathway' which comprises multiple core pro-apoptotic components such as *Bad* (BCL2-associated agonist of cell death), *Bax* (BCL2-associated X protein) and *Bid* (BH3 interacting domain death agonist; Fig. 6a; Supplementary Data 8). Notably, spatiotemporal control of apoptosis plays a particularly crucial role in shaping limb morphogenesis and is required for example, to eliminate webbing between digits where there is a genetic requirement for *Bax*, and *Bak* in the control of this process^24^. Cell death is also required within the anterior and posterior necrotic zones as well as within the opaque patch of the limb bud^25^. Altogether, these findings reveal unique tissue-specific programs of translational control, adding an important layer of regulation that further diversifies the spatial expression of genes between tissues at the same stage of embryonic development.

## Discussion

Altogether, our studies provide the first snapshots of pervasive translational regulation in developing mammalian embryos, which is fundamental to tissue patterning and cell fate specification. Previous studies using synthetic and systems biology approaches have proposed that translation could be a greater source of noise in gene expression than transcription^26^. Therefore, the overall widespread translational regulation identified in our studies may minimize noise and fluctuations to ensure the robustness of embryogenesis in gene expression and act as a gatekeeper of cell signalling critical for developmental decisions. We speculate that the translation control of upstream signalling components and/or key enzymes that are usually dosage-sensitive and rate limiting, may increase the magnitude of translational regulation in efficiently controlling the overall output or strength of a particular signalling pathway. Such highly tunable translation regulation may act as a rapid response to upstream signalling cues and further feedback on control of the pathway. This level of translational regulation to the core signalling circuitry may further explain how the same signalling pathways can be reused in different tissues and time points in development to give rise to distinct outputs in cell fate decisions and tissue patterning. In particular, the unexpected widespread differences in TE observed spatially between different developing tissues offer a tissue-specific programme to selective modulate the levels and magnitude of cell signalling at the post-transcriptional level.

One outstanding question is the nature of both *cis-* and *trans-*acting molecular components that may confer transcript-specific translational regulation to mammalian development of the hundreds of key cell signalling and patterning genes as identified in our studies. Our analysis reveals that the cues for translation regulation are driven, at least in part, by information encoded in the 5′-UTR of the transcript itself in the form of a complex landscape of uORFs. Our mutagenic analysis of uAUGs within signalling components coupled by their CRISPR-mediated deletion in mESCs provides direct evidence for the role of uORF usage in regulation of developmental signalling pathways. Future studies will enable further characterization of additional *cis-*acting elements and eventually to define the repertoire of *trans-*acting factors that might confer spatial and temporal specificity at the level of translation. In this respect, the pronounced effect of uORF-mediated repression on PTCH1 translation and Shh signalling is unexpected and suggests that a default state may be to stringently limit the threshold of pathway activation at the level of translational control. This point of regulation may enable more fine-tuned expression of pathway components beyond transcriptional control and also limit the upper levels in possible activation of key signalling pathways. For example, several components of the Shh signalling pathway when overexpressed can also cause tissue overgrowth and cancer development^27^,^28^,^29^ and thereby stringent translational repression may serve as a critical bottleneck to inappropriate pathway activation.

Altogether, our findings underscore a vital layer of post-transcriptional gene regulation guiding mammalian development and suggest that the translationally regulated profiles of mRNAs, in particular the core signalling components, 5′-UTR *cis*-regulatory elements, and molecular regulators of transcript-specific translational control have endowed a new layer of regulation to a relatively fixed number of patterning and cell signalling genes utilized throughout metazoan evolution.

## Methods

### Mice

Mice were housed under a 12 h light/dark cycle with free access to food and water. *T-Cre* (*Tg(T-cre)1LWD/J*) mice were generously provided by Mark Lewandoski. The *mTmG* (*R26-mTmG*^*flox/flox*^ (Stock#007576)) were purchased from the Jackson Laboratory (Bar Harbor, ME, USA). FVB/NJ (Stock# 001800) mice were purchased from Jackson Laboratory and used as wildtype. All animal work was performed in accordance with protocols approved by Stanford University's Administrative Panel on Laboratory Animal Care.

### FACS sorting

Filming media (DMEM F12 1:1, 10% FBS) and PBS were chilled on ice and treated with 100 μg ml^−1^ cycloheximide (Sigma, 01810). Pregnant *R26-mTmG*^*flox/flox*^ females were crossed with *Tg(T-cre)1LWD/J* males. The morning of vaginal plug was considered as E0. Pregnant females, 3–8 months of age, were euthanized at day E11.5 of gestation and the uterus was removed and washed with 1xPBS containing 100 μg ml^−1^ cycloheximide. Embryos were screened under a fluorescent microscope to identify *R26-mTmG*^*flox/+*^; *Tg(T-cre)1LWD/J* embryos, which were then dissociated into single cells using 1% trypsin (Gibco, 27250-018) containing 100 μg ml^−1^ cycloheximide at 37 °C for 30 min.The reaction was stopped with addition of media followed by pipetting (10 times) to dissociate cells completely. Cells were then washed with PBS, re-suspended in filming media, and passed through a 40-μm nylon filter (Falcon, 352340) to remove doublets and cell clumps. Dissociated cells were FACS sorted into GFP^+^ populations at Stanford Shared FACS Facility. Cells were collected into filming media and washed with PBS. Cells were then split into two tubes for Ribo-Seq and RNA-Seq respectively.

### Dissection of neural tube and forelimb

Pregnant FVB females, 3–8 months of age, were euthanized at E11.5, the uterus was dissected and embryos were taken out and placed into PBS containing 100 μg ml^−1^ cycloheximide. Microdissections were performed in filming media (DMEM F12 1:1, 10% FBS) containing 100 μg ml^−1^ cycloheximide in a Sylgard dissection dish (Sylgard 184 Silicone Elastomer Kit; Dow Corning). The neural tube and forelimbs were collected from the same embryos. For neural tube analysis, embryos were pinned to the dish (Austerlitz dissecting pins, FST) with the ventral surface of the embryo facing down. The neural tubes were separated from somites utilizing a tungsten needle (Sharpoint) from the hind limb, which served as a landmark, to the rhombomeres. For some experiments, neural tubes were snap frozen in liquid nitrogen and stored at −80 °C. Otherwise, they were dissociated at 37 °C for 30 min using Papain tissue dissociation kit (Washington, LK003150). After incubation, cells were washed using manufacture's protocol, rinsed in PBS and then split into two tubes for Ribo-Seq and RNA-Seq respectively.

### Ribo-Seq

For Ribo-Seq, the cell pellet was re-suspended in 200 μl of cold lysis buffer (20 mM Tris pH 7.5, 150 mM NaCl, 15 mM MgCl_2_, 100 μg ml^−1^ cycloheximide, 1 mM DTT, 1% Triton X-100, 8% glycerol, 20 U ml^−1^ TURBO DNase (Ambion, AM1907)) and incubated for 30 min at 4 °C with occasional vortexing. The lysate was clarified by sequential centrifugation for 5 min at 1,800*g* and 10,000*g* at 4 °C to remove nuclei and mitochondria. The lysate was then treated with RNase I (Ambion, AM2294) for 30 min at RT to digest mRNAs not protected by the ribosome. The digestion was stopped by adding 4.5 μl of SUPERaseIn RNase Inhibitor (20 U μl^−1^, Ambion AM2696). Lysate was then loaded onto a 1 M sucrose cushion. Ribosomes were pelleted by ultracentrifugation at 70,000 r.p.m. for 4 hr at 4 °C by TLA120.2 rotor. The pellet was re-suspended in Trizol (Invitrogen, 15596) and RNAs were extracted by following manufacturer's protocol.

### RNA-Seq

After centrifugation, the cell pellet was re-suspended in Trizol (Invitrogen, 15596) for RNA extraction. RNA was extracted and polyA mRNA was isolated using Oligotex mRNA Mini Kit (Qiagen, 70022) following manufacturer's protocol. Purified mRNAs were fragmented in alkaline fragmentation buffer (100 mM NaCO_3_ pH9.2, 2 mM EDTA).

### Deep-sequencing library production

Library preparation was conducted following the published protocol^11^. Briefly, RPFs and fragmented RNAs were loaded onto a 15% urea gel. 28–31 nt RPFs and 30–50 nt fragmented RNAs were excised from the gel for Ribo-Seq and RNA-Seq respectively. RNAs were eluted, dephosphorylated by PNK (NEB, M0201S), and ligated to the miRNA Cloning linker (NEB, S1315S) by T4 RNA Ligase2 truncated K227Q (NEB, M0242S). Ligated RNA was gel purified and reverse transcribed by Superscript III (Invitrogen, 18080). Gel purified cDNAs were circularized by Circligase (Epicentra, CL4111K) and rRNA sequence were subtracted using biotinylated oligos^10^. Amplification was done using Phusion High Fidelity DNA Polymerase (NEB, M0530S). PCR amplification was performed for 10–15 cycles and products were loaded onto non-denaturing 8% PAGE gel. DNA fragment were purified for Illumina sequencing.

### Sequence alignments

Sequencing reads were parsed by cutadapt^30^ to remove the 3′ adapter sequence and reads with good sequencing qualities (Phred quality score>33) were kept. A layered alignment was performed to first discard reads mapping to rRNA, tRNA or snRNA sequences. Non-rRNA/tRNA/snRNA reads were then aligned against the canonical isoform of UCSC known gene transcripts (mm10)^31^. Only uniquely mapped reads were kept for the analysis. The total number of reads, reads after rRNA/tRNA/snRNA removal, and number of unique reads mapped to the canonical isoform of UCSC known genes are reported in Supplementary Data 1.

### Assessing the quality of deep-sequencing libraries

The quality of the Ribo-Seq and RNA-Seq libraries was assessed by analysing the distribution of reads aligning to different functional regions (5′-UTR, CDS and 3′-UTR) of the transcriptome, reads size distribution, triplet periodicity and metagene analysis of reads distribution around the beginning and end of CDS. The set of genes included in the metagene analysis was obtained after filtering by gene length (5′-UTR≥50 nt, 3′-UTR≥50 nt and CDS≥500 nt) and number of reads (≥300 reads mapped to the gene). Reads mapping to each gene were further normalized to the same scale by dividing the total number of reads mapped to that gene. The average reads density near the start (last 30 nt in 5′-UTR and first 150 nt in CDS) and stop (last 150 nt in CDS and first 30 nt in 3′-UTR) across all genes analysed were plotted in Supplementary Fig. 3d.

### Analysis of translational efficiency and regulation

Mapped RPFs were assigned to a specific position based on the A sites. The position of A site in relative to the 5′ end of each read is calculated according to the following rule: ≤29 nt:+14 nt; 30–31 nt:+15; 32–35 nt:+16 and ≥36 nt:+17, similar to previously described^10^. Since translational efficiency is assessed based on the abundance of RPFs compared with the RNA abundance, RNA-Seq reads were processed in the same way as RPFs. Due to the pileup of ribosomes at the beginning of coding region caused by the cycloheximide treatment (Supplementary Fig. 3d), reads mapping to the first 15 or last 5 codons were excluded from the analysis following published methods^10^,^11^. To simplify the analysis, only the canonical isoform of UCSC known gene transcripts (mm10)^31^ was considered. For each gene, total number of RPFs and RNA reads mapping to the CDS excluding the first 15 or last 5 codons were counted and fed into the downstream analysis. The Ribo-Seq and RNA-Seq were analysed together applying the framework of generalized linear model (GLM) in the DESeq R package^12^,^13^. First, the effective library size of each deep-sequencing library was calculated and raw read counts data were normalized by DESeq. Normalized read counts of each biological replicate were reported in Supplementary Data 2 and 7. Variance-mean dependence in count data was estimated and differential gene expression/regulation was tested using a model of negative binomial (NB) distribution. Specifically, for the analysis translation regulation in one tissue type (that is, mesoderm for Fig. 1), a linear regression was performed to the normalized read counts with log link, as a function of library type (‘RNA-Seq' or ‘Ribo-Seq') and replicate variables (‘replicate 1', ‘replicate 2' or ‘replicate 3'). Here the coefficient of library type variable (‘Ribo-Seq' over ‘RNA-Seq') is a measurement of TE. To reveal the differences in translational regulation comparing between tissues (neural tube and forelimb in Fig. 5), we analysed the interaction term of the library types (‘Ribo-Seq' and ‘RNA-Seq') with sample types (‘Neural tube' or ‘Forelimb'). The coefficient of this interaction term is a measurement of changes in TE between tissues. P-values were adjusted using the Benjamini-Hochberg (BH) procedure for multi-testing and these adjusted p-values (that is, FDR) were reported for each gene in Supplementary Data. We used the DREME tool^32^ to search for enriched sequence motifs among translationally regulated components of the Shh, Wnt and Hippo pathways (shown in Fig. 3a). Shuffled input sequence was used as control. No significantly enriched sequence motifs were identified in these 5′-UTRs.

### GO and pathway analysis

Gene ontology (GO) enrichment and clustering analysis was performed using ClueGO^33^ for genes under translational control in the mesoderm at E11.5 (Fig. 1), and genes with distinct TE between the neural tube and forelimb (Fig. 6). The following parameters were used in all analyses: Reference set: all genes analysed; Enrichment: Right-sided hypergeometric test; *P*-value adjustment: Benjamini-Hochberg adjustment. Enriched GO terms were then grouped using Kappa statistics with leading group term based on highest statistical significance. For Fig. 1c, each group is displayed by different colour-shaded circles. Each node represents one significantly enriched GO term colour-coded by its adjusted p-value (FDR) of enrichment, with size of the node proportional to the number of associated genes. Edges indicate similarity (Kappa score >0.4) between the two connected GO terms. Edges with an arrowhead indicate a regulation relationship between nodes. For enriched GO categories of genes with distinct TE between the neural tube and forelimb, only the leading GO terms in each group with adjusted *P*<0.05 and containing ≥10 genes are displayed in Fig. 6a, and all enriched GO terms with adjusted *P*<0.05 are listed in Supplementary Data 8.

### 5′ RACE

RNA was purified from dissected E11.5 neural tubes and somites using Trizol (Invitrogen, 15596). Full-length status of the transcript was defined based on the presence of a capped 5′ end. The reverse transcription template was produced using ExactSTART Eukaryotic mRNA 5′-& 3′-RACE Kit (Epicentre, ES80910) following the manufacturer's protocol and reverse transcribed using Superscript III with a primer in the coding region of *Ptch1*, *Smo*, *Gli1, Wwc1, and Sav1*. For each gene, the 5′-UTR fragment was amplified using KOD Xtreme Hot Start DNA polymerase (Millipore, 71975) with a gene specific reverse primer and a forward primer at the 5′ linker. PCR products were adenylated, gel extracted and subcloned into TOPO TA cloning vector (Invitrogen, 450030) for sequencing.

### Plasmids

Identified 5′-UTRs were amplified and cloned into the HindIII and NcoI sites of pGL3 –SV40 control vector (Promega) using Gibson assembly (NEB, E2611). Mutations of upstream start sites, changed to AAG or CAG, were generated by site-directed mutagenesis. Non-canonical start sites with a favourable Kozak sequence were defined as A/G at +3 nt and G at −1 nt position. The stop codon (ugUGA) for *Ptch1* 3rd and 5th uAUGs was mutated to ugAGA. This resulted in a new STOP codon for the 4th uAUG. To make frame shifts, C or CC were inserted just before the main CDS. The 5′-UTR sequences are presented in Supplementary Table 1 and all plasmids are listed in Supplementary Table 2.

### Fluc and Rluc activity

NIH3T3 cells were seeded in 12-well plates at a density of 1 × 10^5^ cells per well in growth media (10% FBS (Hyclone, SH30071.03), Penn/Strep (Gibco, 15140–122), DMEM (Gibco, 11965–118). Cells were transfected with 800 ng of pGL3 (Firelfy luciferase) construct containing full-length or mutant 5′-UTRs of Shh or Hippo pathway components or HBB and 100 ng of pRL (Renilla luciferase) control construct per well, with Lipofectamine 2,000 in Optimem (Invitrogen, 11668-019; 11058-021) following manufacturer's instructions. At 48 h post-transfection, cells were washed twice with PBS, harvested and split between two tubes. Cells in one tube were suspended in Trizol (Invitrogen, 15596) and RNA was purified using PureLink RNA Mini Kit (Thermo Fisher, 12183018) followed by TURBO DNase (Ambion, AM1907) treatment. Firefly and Renilla luciferase RNA amounts were quantified by RT-qPCR. The cells in the second tube were lysed and assayed using Dual Luciferase kit (Promega, E1980). Firefly luciferase activity was normalized to Renilla luciferase activity and the translation efficiency of Firefly reporter construct was calculated from Firefly luciferase RNA amount normalized to Renilla luciferase relative to the *HBB* construct. Each experiment was performed a minimum of three times, each containing two technical replicates. Statistical analysis was performed using Student's *t*-test.

### CRISPR/Cas9-mediated deletion and neuronal differentiation

CRISPR/Cas9-mediated deletion of uORFs in Ptch1 5′-UTR was performed largely as described by Li *et al*.^34^. In brief, guide RNAs (gRNAs) were designed using the CRISPR design tool ( http://crispr.mit.edu/) - three individual gRNAs were designed for cleavage on the 5′ and on the 3′ side of the targeted region and tested for efficient deletion of the target region in mouse ESCs. Each gRNA was subcloned into plasmid encoding Cas9 with an integrated puromycin selection cassette ( https://www.addgene.org/62988/). Pairs of gRNAs for the *Ptch1* uORF deletion were transfected into *Nkx2.2:CreEGFP* mESCs (a kind gift of Drs Lori Sussel and Mark Magnuson): cells were seeded at a density of 500,000 cells per well of a gelatinized 6-well plate in standard ES cell media 15% FBS (Millipore, ES-009-B), 1 × L-glutamine (TMS-002-C, Millipore), 1 × non-essential amino acids (TMS-001-C, Millipore), 300 μl 2-mercaptoethanol (ES-007-E, Millipore), 30 μl LIF (ESG1107, Millipore) in 250 ml Knockout DMEM (10829-018, Life Technologies), 1 × penicillin/streptomycin (TMS-AB2-C, Millipore) supplemented with 1 mM MEK inhibitor (Cayman, 13034) and 1 μM GSK3 inhibitor (CHIR99021, Cayman, 13122). Cells were allowed to adhere for 4 h, media was changed and 30 min later cells were transfected using Lipofectamine 3,000 (Invitrogen, L3000001) following manufacturer's instructions. For each well, we used 1 μg of each gRNA as well as 0.5 μg of membrane Kate plasmid to allow monitoring of transfection efficiency. Media was changed after 4–6 h. Cells underwent selection 24 h after transfection using 1.5 μg ml^−1^ puromycin for 48 h (Sigma, P8833-10MG). The surviving cells were seeded at low density on gelatinized plates and 4–7 days after plating individual clones were picked and replica plated onto two gelatinized 96-well plates. After 4–7 days, homozygous mutants were identified by positive PCR genotyping to identify presence of the mutant allele (using primers flanking the deleted region) and confirmed by negative PCR to confirm absence of wildtype allele (using a primer within the deleted region). Homozygous clones were expanded and used for the differentiation experiments. PCR primers and gRNA sequences are listed below:

*Ptch1* F 1348 5′-CGGCGTTACCAGCCGAGGCC-3′

*Ptch1* R 1671 5′-CTGCTGGTGCTGCTGCGGCT-3′

*Ptch1* Internal 1670 5′-GCCTTCCATTGCCACATTGCG-3′

gRNA *Ptch1* 67 5′-GCGGGTCTGTCACCCGGAGC CGG-3′

gRNA *Ptch1* 65 5′-GCGCGGGGCGGGGACGTCTG GGG-3′

### Monolayer NPC differentiation

Mutant and control mESCs were differentiated into ventral motor neurons following protocols established by E. Kutejova and J. Briscoe^19^. Briefly, CellBind tissue culture dishes (Corning, 3294) were gelatinized overnight in 0.1% gelatin solution (Sigma, G1393-100 ml) and washed twice with 1 × PBS the following day. Mutant and control wildtype mESCs were trypsinized following standard protocols and pelleted in fresh mESC media. The pellets were washed twice (first in 1xPBS and then in N2B27 media) and the cells were re-suspended in 1 ml of fresh N2B27 media (DMEM/F12+Glutamax Invitrogen, 10565-018, Neurobasal medium (Invitrogen, 21103-049), B27 (Invitrogen, 17504-044), N2 Max media supplement (R&D, AR009), BSA 20 mg ml^−1^ in PBS (Sigma, A3156), penicillin/streptomycin (TMS-AB2-C, Millipore), B-mercaptoethanol (M3148-25ML, Sigma), counted and a drop of 75,000-85,000 cells in 100 μl of media was seeded in the center of each dish. Cells were allowed to settle for 3 min and the dishes were topped up with 1.5 ml of N2B27 medium. Media was changed every 24 h from day 0 to day 3. On day 3, the media was supplemented with 30 nM Retinoic Acid (R2625-100MG, Sigma). After 8 h of culture, fresh media containing 30 nM RA and either 500 nM SAG (5666601-1 mg, Millipore/Calbiochem) or 2 μg ml^−1^ of recombinant SHH protein (464-SH-025, R&D) was added^19^,^30^- this was counted as day 3.5. Between day 3.5 and day 5 media (supplemented with fresh RA and either SAG or SHH protein) was changed every 12 h. Cells were collected at 0, 4, 12 h or day 5 (36 h) after the initiation of SAG or SHH treatment as indicated and used for downstream analysis. For immunostaining, cells were washed 3 × with cold 1 × PBS and fixed for 10 min in 4% PFA/PBS at 4 °C. Cells were then washed 3 × with cold 1xPBS and incubated for 1 h in blocking solution (10% goat serum, 0.1% Triton-X in PBS) and incubated with primary antibodies against FOXA2 (1:100, DSHB, 4C7c) or NKX2.2 (1:100, DSHB, 74.5A5) in blocking solution, overnight at 4 °C, in a humidified chamber. The next day, the cells were washed in wash buffer (1% goat serum, 0.1% Triton-X, PBS) 3 × and incubated with secondary antibody at a concentration of 1:200 in blocking solution (goat-anti-mouse Alexa 488 IgG, Invitrogen, A11017) and DAPI (1:1,000) for 45 min at room temperature. Cells were washed with wash buffer 3 × , mounted in Fluoromount-G (SouthernBiotech) and examined using a Spinning Disc confocal microscope. For each mutant or control line, we examined 3–5 independent cultures seeded on different days. For each sample, we acquired 10 images of a single plane at × 63 and quantified the number of positive cells in the image normalized by the surface area containing in-focus, confluent cells. The average number of positive cells/area was determined across 10 images and used as *n*=1. The same analysis was repeated across two other independent samples per genotype. The average number of positive cells/area was then determined for *n*=3 wildtype and *n*=3 mutant samples and compared using Student's *t*-test, <0.05 was deemed significant.

### Western blot analysis

Microdissected neural tubes were lysed in 50 μl RIPA buffer (150 mM NaCl, 50 mM Tris-HCl (pH7.4), 5 mM EDTA (pH8.0), 1 mM EGTA (pH8.0), 0.1% SDS, 1.0% NP-40, 0.5% deoxycholate, 1 × Combined Protease and Phosphatase Inhibitor (Thermo 78443)) and 10 μl of the lysate was removed and dissolved in Trizol (Invitrogen, 15596) for RNA extraction and RT-qPCR analysis. Lysates were incubated for 5 min on ice and genomic DNAs were sonicated by Bioruptor (Diagenode). Cell lysates were then spun at 14,000 r.p.m. for 5 min at 4 °C to remove debris and the supernatant was collected. For western blot analysis, protein concentration was measured by BCA assay (Pierce, 23225), and 20 μg of protein was loaded onto 4–20% SDS–PAGE gel. After running, proteins were transferred by semi-dry transfer system using Trans-Blot Turbo (Bio-Rad) following manufacture's protocol. PTCH1 proteins were wet transferred to a PVDF membrane using a Mini Trans-Blot Electrophoretic Transfer Cell (Bio-Rad). The PVDF membranes were blocked in 5% nonfat dry milk in PBST for 1 h, and incubated overnight at 4 °C with anti-PTCH1 (1:500, kind gift of Matthew Scott's lab), anti-ACTB (1:5,000, Sigma) and anti-GAPDH (1:5,000, Ambion, AM4300), then washed three times for 5 min in PBST, incubated with appropriate secondary antibodies conjugated to horseradish peroxidase (anti-Mouse and anti-Rabbit from GE Healthcare) for 1 h, and then washed three times for 5 min in PBST. The western blot signals were developed using Clarity Western ECL Substrate (Bio-Rad, 1705060) and imaged with ChemiDoc MP (Bio-Rad). Signal intensity of each band was quantified by ImageJ and protein amount was normalized to GAPDH and compared between tissues or genotypes.

### RT-qPCR analysis

For embryonic RNA preparation, E11.5 neural tubes or forelimb samples were dissected and RNA was isolated with Trizol (Invitrogen, 15596) following the manufacturer's protocol. 0.1 μg of RNA (both of total and sucrose-gradient sample) was converted to cDNA using iScript Reverse Transcription Supermix for RT-qPCR (BioRad, 1708840). cDNA was diluted twofold and 2 μl used to run a SYBR green detection RT-qPCR assay (SsoAdvanced Universal SYBR Green supermix (1725270) and CFX384, BioRad). Data was analysed and converted to relative RNA quantity using CFX manager (BioRad). Primers were used at 300 nM per reaction. All qPCR primer sequences are listed in Supplementary Table 3.

### Sucrose-gradient fractionation

Sucrose gradient fractionation was performed on microdisected neural tubes or forelimbs from E11.5 (23 +/−2.5 somites from tail to hind limb) following established protocols as described elsewhere^35^. Each embryo was suspended in lysis buffer (20 mM Tris pH 7.5, 150 mM NaCl, 15 mM MgCl_2_, 100 μg ml^−1^ cycloheximide, 1 mM DTT, 1% Triton X-100, 8% glycerol, 20 U ml^−1^ TURBO DNase (Ambion, AM1907), 100 U ml^−1^ SUPERase RNase inhibitor (Ambion)), dissociated by pipetting 10 times and incubated on ice for 30 min. After fractionation, 100 ul of 10% SDS (final concentration 1%) containing 1 ng ml^−1^ *in vitro* transcribed Firefly luciferase RNA was added to each fraction and RNAs were extracted using Phenol/chloroform followed by isopropanol precipitation and fractions were combined for RT-qPCR. RNA purification efficiency for each fraction was normalized by Firefly luciferase RNA.

### Data availability

Sequencing data are deposited in the Gene Expression Omnibus under accession number GSE86467. 5′UTR sequences have been deposited in Genbank, under accession numbers KY442312 (*Ptch1*), KY442313 (*Smo*), KY442314 (*Gli1*), KY442315 (*Nf2*), KY442316 (*Wwc1*), KY442317 (*Sav1*), and KY442318 (*Lats2*). The data that support the findings of this study are available from the corresponding author upon request.

## Additional information

**How to cite this article:** Fujii, K. *et al*. Pervasive translational regulation of the cell signalling circuitry underlies mammalian development. *Nat. Commun.* **8,** 14443 doi: 10.1038/ncomms14443 (2017).

**Publisher's note**: Springer Nature remains neutral with regard to jurisdictional claims in published maps and institutional affiliations.

## Supplementary Material

Supplementary Information Supplementary Figures and Supplementary Tables

Supplementary Data 1 Sequencing depth and reads distributions of the Ribo-Seq and RNA-Seq libraries

Supplementary Data 2 The transcriptional and translational profiles of gene expression in the mesoderm at E11.5

Supplementary Data 3 Enriched GO categories (biological processes) among TE-high genes in the mesoderm at E11.5

Supplementary Data 4 Enriched GO categories (biological process) among TE-low genes in the mesoderm at E11.5

Supplementary Data 5 Enriched GO categories (molecular function) among TE-low genes in the mesoderm at E11.5

Supplementary Data 6 The transcriptional and translational profiles of Fig. 2 signaling components in the mesoderm at E11.5

Supplementary Data 7 RNA-Seq and Ribo-Seq identified genes with significantly different TE comparing the neural tube (NT) and forelimb (FL) at E11.5

Supplementary Data 8 Enriched GO categories (biological process) among genes with significantly different TE in the neural tube (NT) compared to the forelimb (FL) at E11.5

## References

[b1] Shi Z. & Barna M. Translating the genome in time and space: specialized ribosomes, RNA regulons, and RNA-binding proteins. Annu. Rev. Cell Dev. Biol. 31, 100814–125346 (2015). 10.1146/annurev-cellbio-100814-125346 26443190

[b2] Sonenberg N. & Hinnebusch A. G. New modes of translational control in development, behavior, and disease. Mol. Cell 28, 721–729 (2007). 18082597 10.1016/j.molcel.2007.11.018

[b3] Livingstone M., Atas E., Meller A. & Sonenberg N. Mechanisms governing the control of mRNA translation. Phys. Biol. 7, 21001 (2010). 10.1088/1478-3975/7/2/021001 20463379

[b4] Thompson B., Wickens M. & Kimble J. in Translational Control in Biology and Medicine Vol. 48, (eds Mathews, M. B., Sonenberg, N. & Hershey, J.W.B.) 507–544Cold Spring Harbor Laboratory Press (2007).

[b5] Bazzini A. A., Lee M. T. & Giraldez A. J. Ribosome profiling shows that miR-430 reduces translation before causing mRNA decay in zebrafish. Science 336, 233–237 (2012). 22422859 10.1126/science.1215704 PMC3547538

[b6] Li L., Zheng P. & Dean J. Maternal control of early mouse development. Development 137, 859–870 (2010). 20179092 10.1242/dev.039487 PMC2834456

[b7] Ingolia N. T., Ghaemmaghami S., Newman J. R. S. & Weissman J. S. Genome-wide analysis *in vivo* of translation with nucleotide resolution using ribosome profiling. Science 324, 218–223 (2009). 19213877 10.1126/science.1168978 PMC2746483

[b8] Muzumdar M. D., Tasic B., Miyamichi K., Li L. & Luo L. A global double-fluorescent Cre reporter mouse. Genesis. 45, 593–605 (2007). 17868096 10.1002/dvg.20335

[b9] Perantoni A. O. . Inactivation of FGF8 in early mesoderm reveals an essential role in kidney development. Development 132, 3859–3871 (2005). 16049111 10.1242/dev.01945

[b10] Ingolia N. T., Lareau L. F. & Weissman J. S. Ribosome profiling of mouse embryonic stem cells reveals the complexity and dynamics of mammalian proteomes. Cell 147, 789–802 (2011). 22056041 10.1016/j.cell.2011.10.002 PMC3225288

[b11] Ingolia N. T., Brar G. A., Rouskin S., McGeachy A. M. & Weissman J. S. The ribosome profiling strategy for monitoring translation *in vivo* by deep sequencing of ribosome-protected mRNA fragments. Nat. Protoc. 7, 1534–1550 (2012). 22836135 10.1038/nprot.2012.086 PMC3535016

[b12] Anders S. & Huber W. Differential expression analysis for sequence count data. Genome Biol. 11, R106 (2010). 20979621 10.1186/gb-2010-11-10-r106 PMC3218662

[b13] Sen N. D., Zhou F., Ingolia N. T. & Hinnebusch A. G. Genome-wide analysis of translational efficiency reveals distinct but overlapping functions of yeast DEAD-box RNA helicases Ded1 and eIF4A. Genome Res. 25, 1196–1205 (2015). 26122911 10.1101/gr.191601.115 PMC4510003

[b14] Ivanov I. P., Loughran G. & Atkins J. F. uORFs with unusual translational start codons autoregulate expression of eukaryotic ornithine decarboxylase homologs. Proc. Natl Acad. Sci. USA 105, 10079–10084 (2008). 18626014 10.1073/pnas.0801590105 PMC2481320

[b15] Bazzini A. A. . Identification of small ORFs in vertebrates using ribosome footprinting and evolutionary conservation. EMBO J. 33, 981–993 (2014). 24705786 10.1002/embj.201488411 PMC4193932

[b16] Chew G.-L., Pauli A. & Schier A. F. Conservation of uORF repressiveness and sequence features in mouse, human and zebrafish. Nat. Commun. 7, 11663 (2016). 27216465 10.1038/ncomms11663 PMC4890304

[b17] Hinnebusch A. G., Ivanov I. P. & Sonenberg N. Translational control by 5 ′ -untranslated regions of eukaryotic mRNAs. Science 352, 1413–1416 (2016). 27313038 10.1126/science.aad9868 PMC7422601

[b18] Mueller P. P. & Hinnebusch A. G. Multiple upstream AUG codons mediate translational control of GCN4. Cell 45, 201–207 (1986). 3516411 10.1016/0092-8674(86)90384-3

[b19] Kutejova E., Sasai N., Shah A., Gouti M. & Briscoe J. Neural progenitors adopt specific identities by directly repressing all alternative progenitor transcriptional programs. Dev. Cell 36, 639–653 (2016). 26972603 10.1016/j.devcel.2016.02.013 PMC4819439

[b20] Gouti M. . *In vitro* generation of neuromesodermal progenitors reveals distinct roles for wnt signalling in the specification of spinal cord and paraxial mesoderm identity. PLoS Biol. 12, e1001937 (2014). 25157815 10.1371/journal.pbio.1001937 PMC4144800

[b21] Gouti M., Metzis V. & Briscoe J. The route to spinal cord cell types: a tale of signals and switches. Trends Genet. 31, 1–8 (2015). 25823696 10.1016/j.tig.2015.03.001

[b22] McManus C. J., May G. E., Spealman P. & Shteyman A. Ribosome profiling reveals post-transcriptional buffering of divergent gene expression in yeast. Genome Res. 24, 422–430 (2014). 24318730 10.1101/gr.164996.113 PMC3941107

[b23] Khan Z. . Primate transcript and protein expression levels evolve under compensatory selection pressures. Science 342, 1100–1104 (2013). 24136357 10.1126/science.1242379 PMC3994702

[b24] Lindsten T. . The combined functions of proapoptotic Bcl-2 family members Bak and Bax are essential for normal development of multiple tissues. Mol. Cell 6, 1389–1399 (2000). 11163212 10.1016/s1097-2765(00)00136-2 PMC3057227

[b25] Zuzarte-Luis V. & Hurle J. M. Programmed cell death in the embryonic vertebrate limb. Semin. Cell Dev. Biol. 16, 261–269 (2005). 15797836 10.1016/j.semcdb.2004.12.004

[b26] Ozbudak E. M., Thattai M., Kurtser I., Grossman A. D. & van Oudenaarden A. Regulation of noise in the expression of a single gene. Nat. Genet. 31, 69–73 (2002). 11967532 10.1038/ng869

[b27] Milenkovic L., Goodrich L. V., Higgins K. M. & Scott M. P. Mouse patched1 controls body size determination and limb patterning. Development 126, 4431–4440 (1999). 10498679 10.1242/dev.126.20.4431

[b28] Hui C.-C. & Angers S. Gli proteins in development and disease. Annu. Rev. Cell Dev. Biol. 27, 513–537 (2011). 21801010 10.1146/annurev-cellbio-092910-154048

[b29] Matise M. P. & Joyner A. L. Gli genes in development and cancer. Oncogene 18, 7852–7859 (1999). 10630638 10.1038/sj.onc.1203243

[b30] Martin M. Cutadapt removes adapter sequences from high-throughput sequencing reads. EMBnet.journal 17, 10–12 (2011).

[b31] Hsu F. . The UCSC known genes. Bioinformatics 22, 1036–1046 (2006). 16500937 10.1093/bioinformatics/btl048

[b32] Bailey T. L. DREME: motif discovery in transcription factor ChIP-seq data. Bioinformatics 27, 1653–1659 (2011). 21543442 10.1093/bioinformatics/btr261 PMC3106199

[b33] Bindea G. . ClueGO: a Cytoscape plug-in to decipher functionally grouped gene ontology and pathway annotation networks. Bioinformatics 25, 1091–1093 (2009). 19237447 10.1093/bioinformatics/btp101 PMC2666812

[b34] Li K., Wang G., Andersen T., Zhou P. & Pu W. T. Optimization of genome engineering approaches with the CRISPR/Cas9 system. PLoS ONE 9, e105779 (2014). 25166277 10.1371/journal.pone.0105779 PMC4148324

[b35] Xue S. . RNA regulons in Hox 5′ UTRs confer ribosome specificity to gene regulation. Nature 517, 33–38 (2015). 25409156 10.1038/nature14010 PMC4353651

