# Supplementary material for: Pervasive translational regulation of the cell signalling circuitry underlies mammalian development
Source: Nat Commun. 2017 Feb 14;8:14443. doi: 10.1038/ncomms14443 (PMC5316868; doi:10.1038/ncomms14443)
Supplement: Supplementary Information — Supplementary Figures and Supplementary Tables [file ncomms14443-s1.pdf]

Supplementary Figures 1-12 and Supplementary Figure legends,
Supplementary Tables 1-3

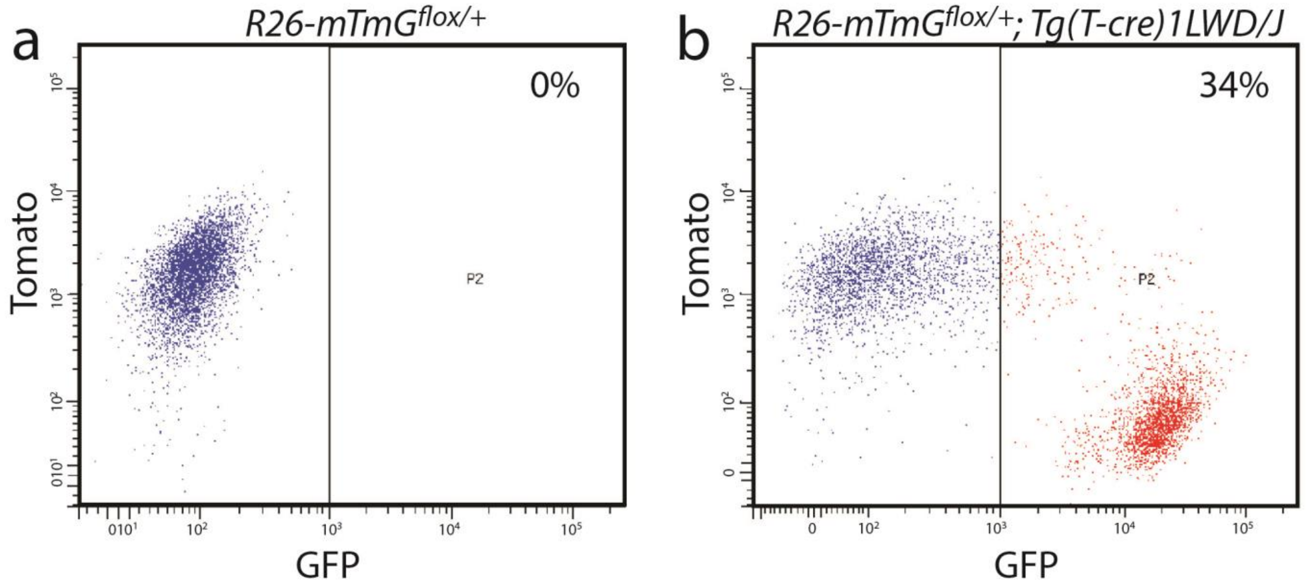

Supplementary Figure 1: Isolation of T-Cre activated Green Fluorescent Protein (GFP) positive cells by Fluorescence Activated Cell Sorting (FACS).
(a) In the *R26-mTmG*$^{flox/+}$ (Cre negative control) embryo, membrane-bound tandem dimer Tomato (mT) is ubiquitously expressed in all cells and we do not detect expression of membrane-targeted enhanced GFP (mG). **(b)** T-Cre activation in *R26-mTmG*$^{flox/+}$; *Tg(T-cre)1LWD/J* embryos results in activation of mG. Both the $mG^+;mT^+$ and $mG^+;mT^-$ populations are detected and collected by FACS ($mG^-;mT^+$ cells do not express T-Cre and are excluded from analysis).

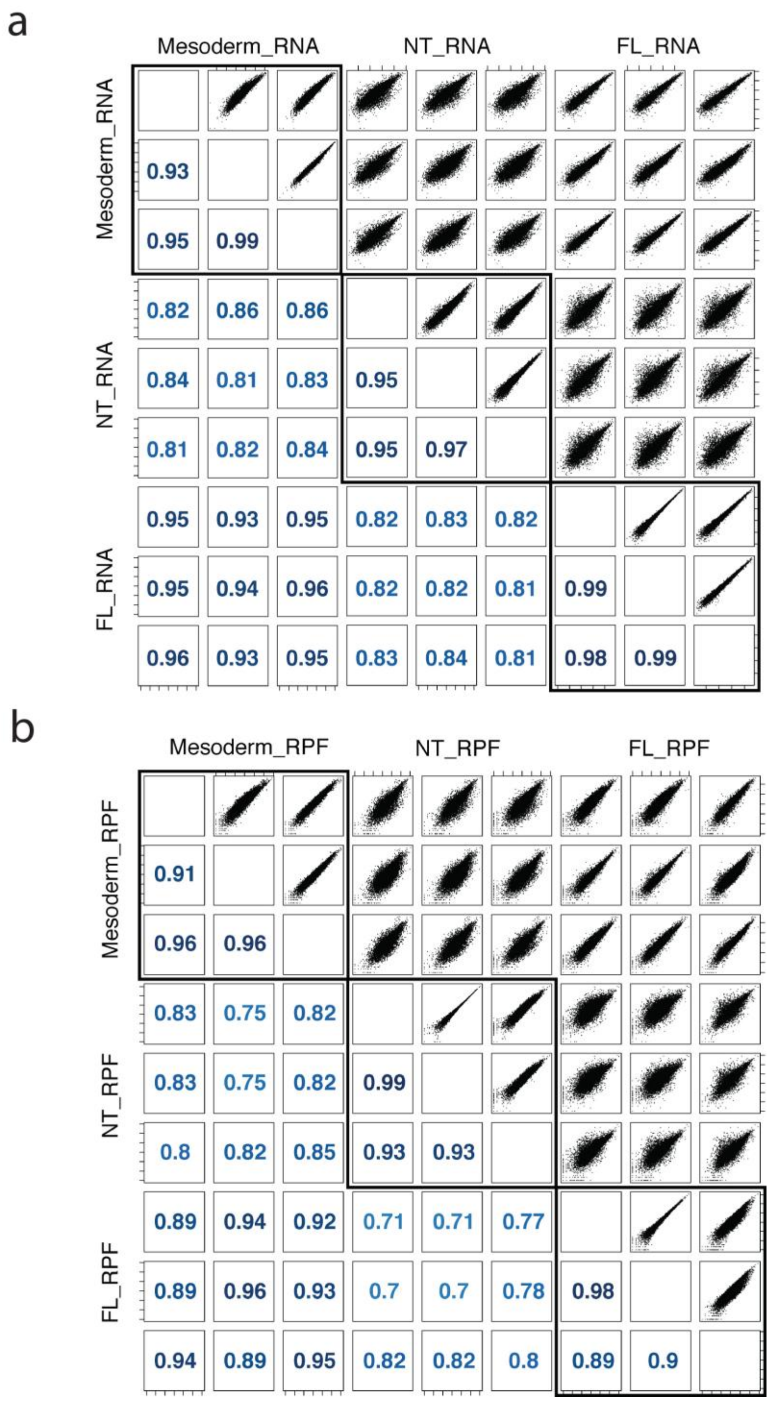

Supplementary Figure 2: Plot of pairwise correlations of all RNA-Seq and Ribo-Seq datasets confirms the high consistency of biological replicates.

Shown are pairwise correlations of all RNA-Seq **(a)** and Ribo-Seq **(b)** datasets. In the upper right corner are the scatter plots and in the bottom left corner are Pearson’s correlations between every pairwise comparison. The correlations between biological replicates along the diagonal (each set of samples with three biological replicates is highlighted in a square) have the highest correlations, confirming the consistency of the dataset.

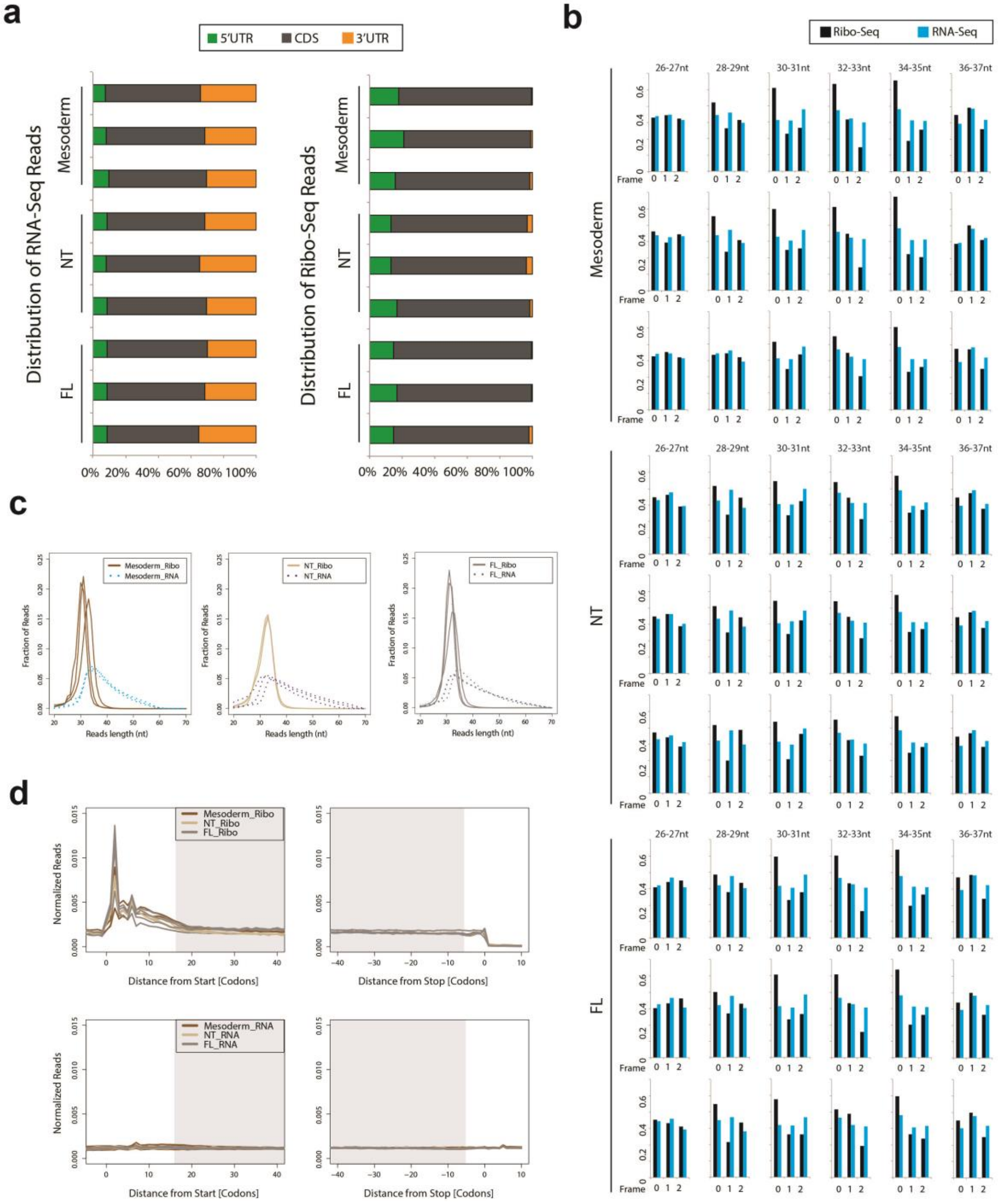

Supplementary Figure 3: Global characterization of the RNA-Seq and Ribo-Seq datasets illustrates the quality of deep sequencing libraries.

(a) Bar graphs show the distribution of RNA-Seq and Ribo-Seq reads mapping to the 5'UTR, CDS and 3'UTR. **(b)** The 3nt periodicity was observed in the Ribo-Seq reads with a length of ~30 nt. RNA-Seq reads do not exhibit the 3nt periodicity. **(c)** Length distribution of RNA-Seq and Ribo-Seq reads showing the Ribo-Seq libraries exhibit a much narrower distribution with the majority of reads being ~30 nt. **(d)** Metagene analysis of read distribution around the beginning and end of CDS of all genes analyzed. Accumulation of RPFs at the beginning of coding region was observed, plausibly caused by the cycloheximide treatment. Therefore, total number of RPFs and RNA reads mapping to the CDS excluding the first 15 or last 5 codons (region in grey) were counted and fed into the analysis of translational regulation, following published methods.

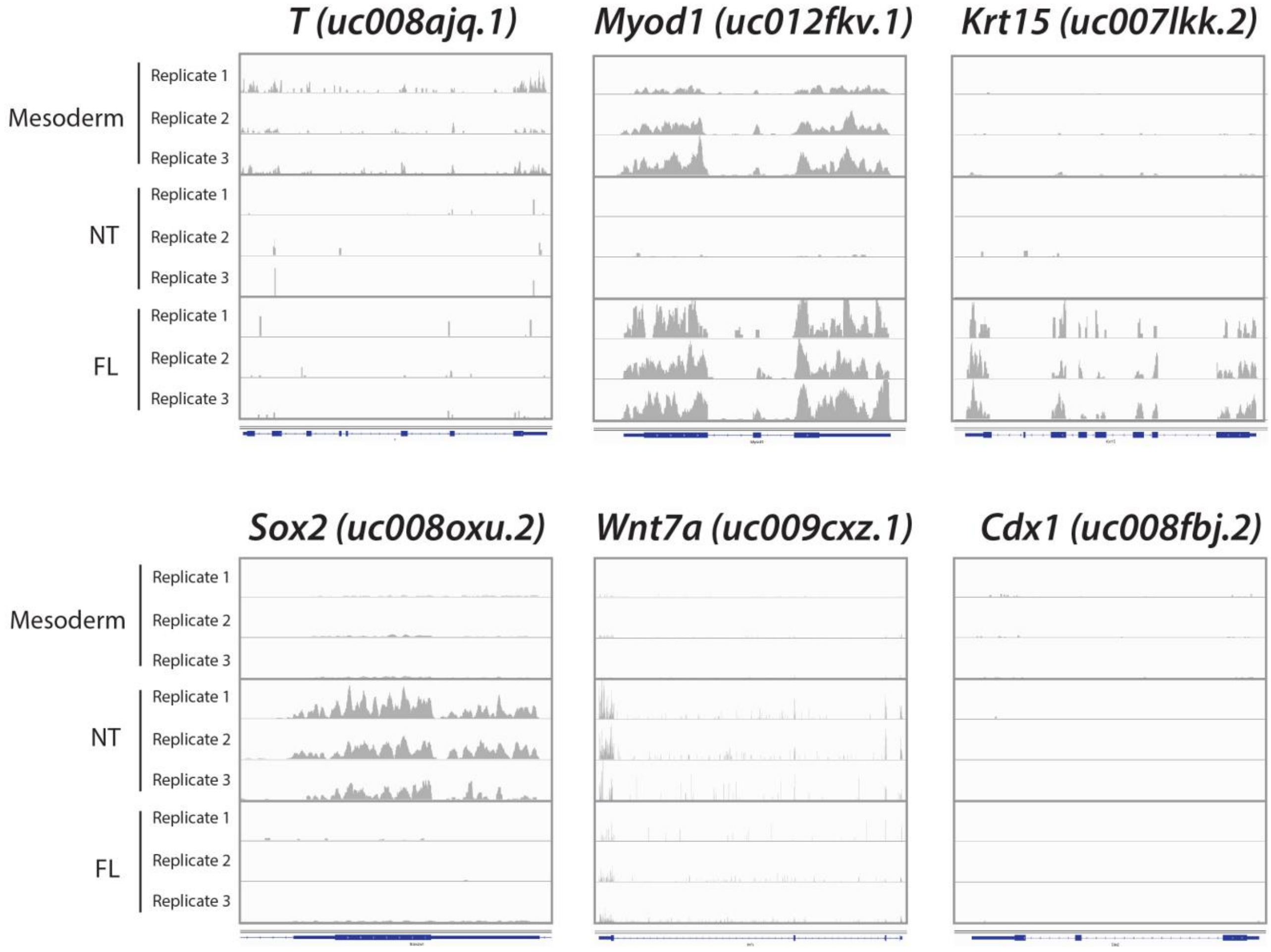

Supplementary Figure 4: Lineage specific marker gene expression reflects quality of isolation of desired cell population.

Shown are the RNA-Seq plots for several well-known lineage specific markers. FACS-isolated mesoderm cells specifically express mesoderm cell markers *T* and *Myod1*; the forelimb expresses mesoderm marker *Myod1* and ectoderm marker *Krt15* and the neural tube expresses markers of neuronal cell fate: *Sox2* and *Wnt7a*. The lack of detectable expression of endoderm cell marker *Cdx1* further ensures the purity of the isolation of desired cell population.

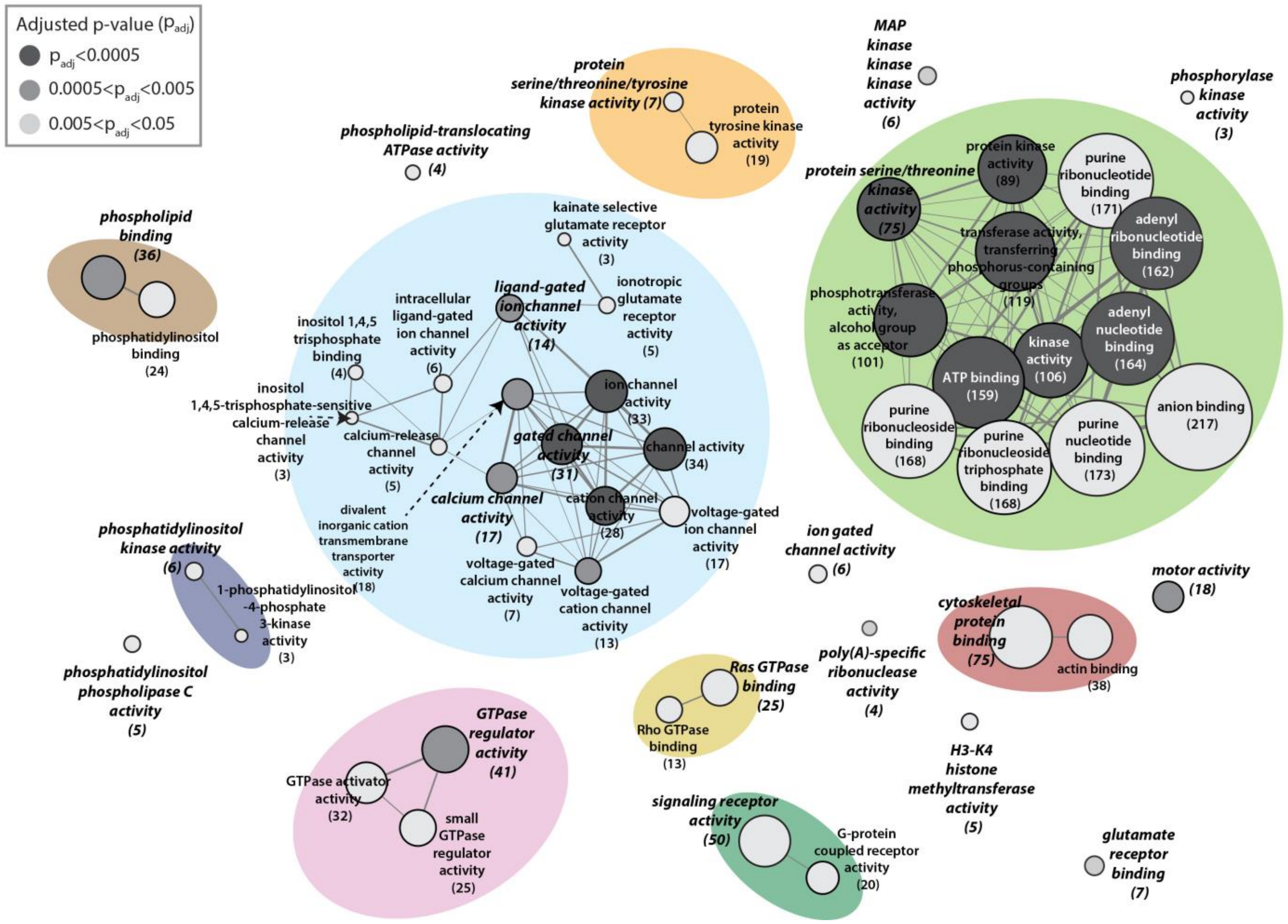

Supplementary Figure 5: The network of enriched Gene Ontology (GO) categories (molecular function) among TE-low genes in mesoderm at E11.5.

The GO terms cluster into several functional groups represented by different color-shaded circles. Each node represents one enriched GO term color-coded by its adjusted p-value (FDR) of enrichment, with size of the node proportional to the number of associated TE-low genes. Edges indicate similarity (Kappa score >0.4) between the two connected GO terms. The number of TE-low genes is shown in the parenthesis following the name of each GO term. The most significantly enriched GO term from each group is designated as the leading group term and highlighted in bold italic.

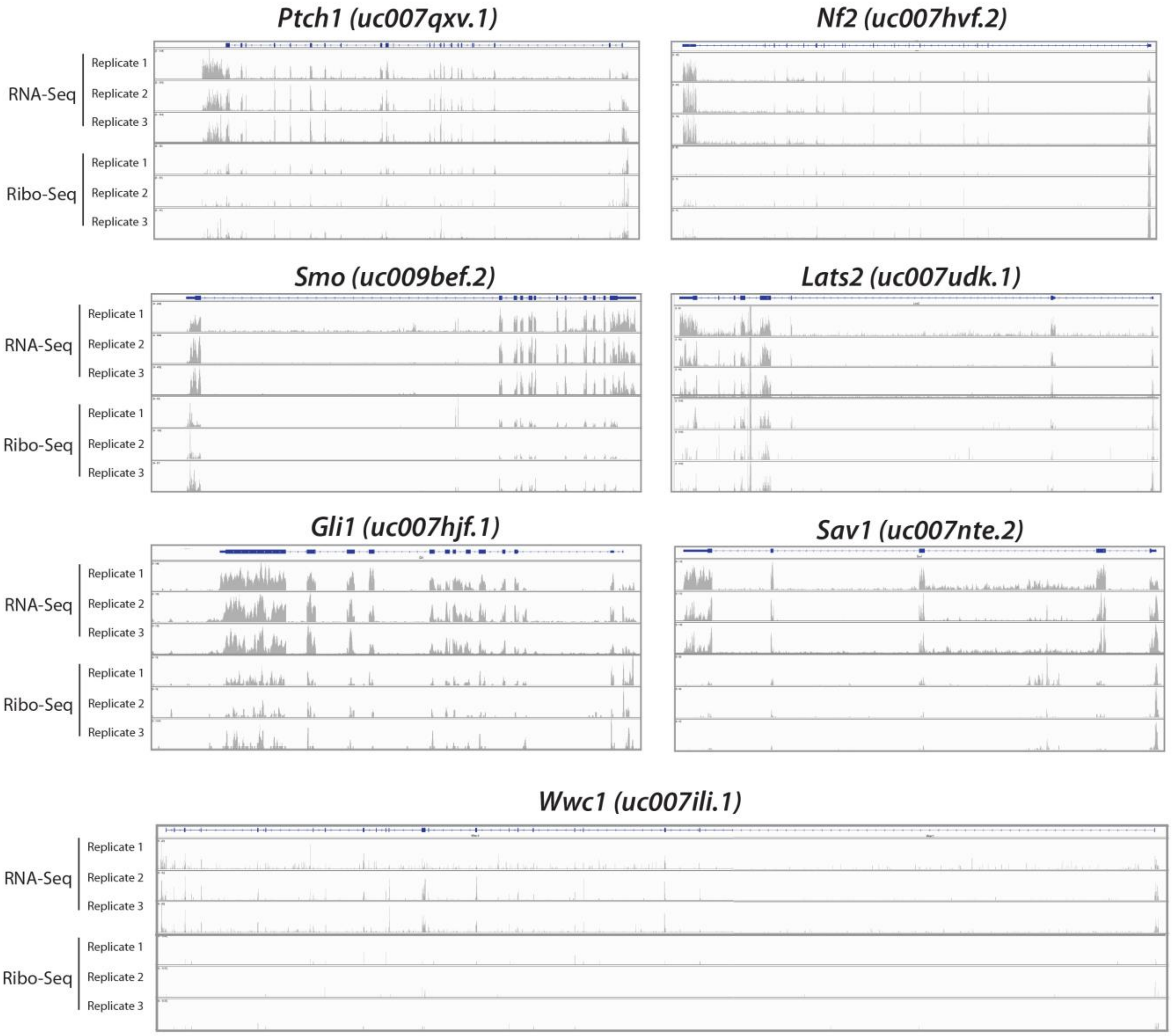

Supplementary Figure 6: RNA-Seq and Ribo-Seq plots of TE-low genes in the Shh and Hippo pathway.

Shown are the E11.5 mesoderm RNA-Seq and Ribo-Seq plots for several Shh and Hippo pathway genes with low TE (analyzed in Figure 3). Majority of reads map to exons (noting that the read density peak in the *Lats2* intron 4 and *Sav1* intron 2 are from the opposite strand).

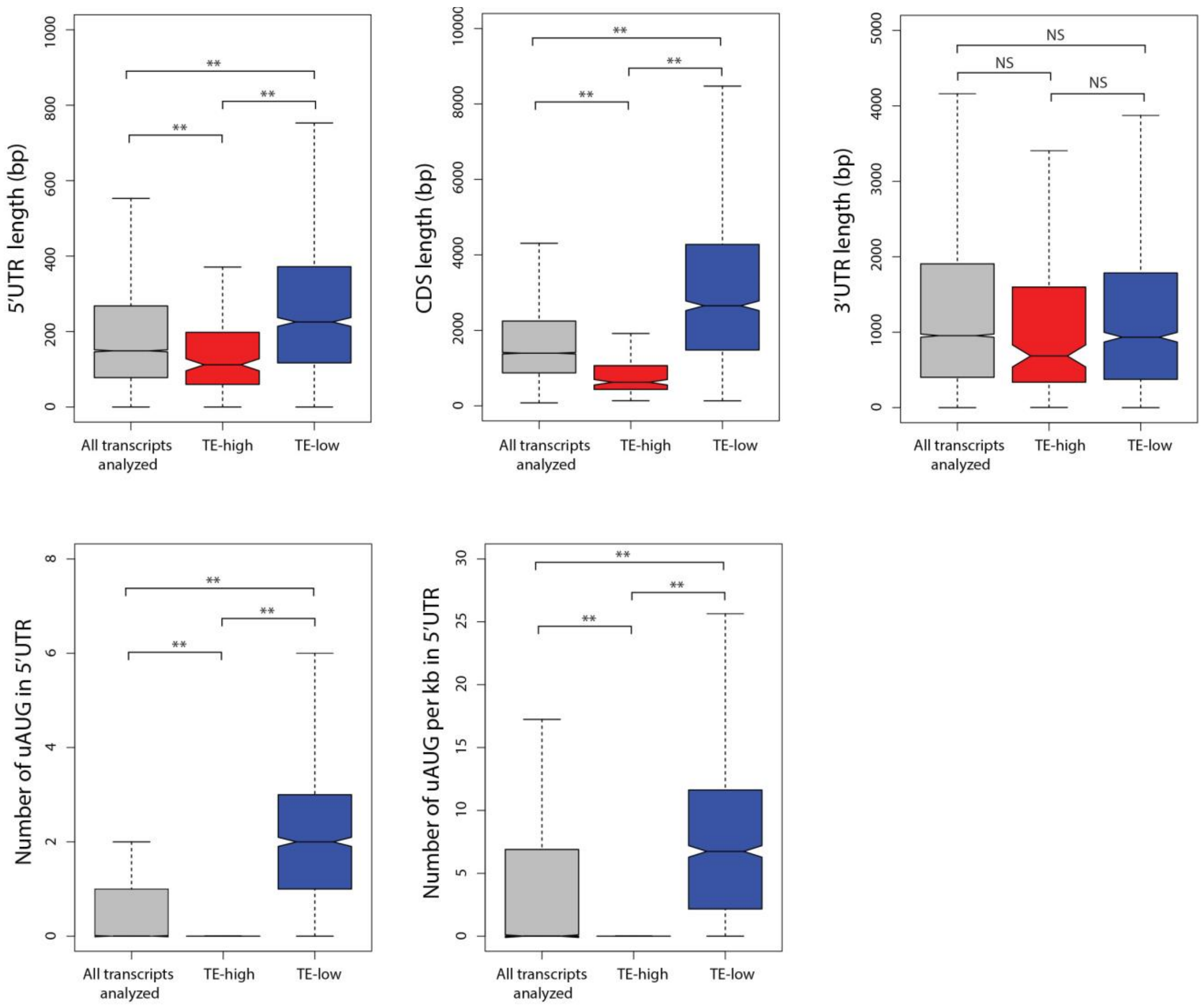

Supplementary Figure 7: General features of translationally regulated transcripts.
Compared to all transcripts analyzed, TE-low transcripts (blue) overall have longer 5'UTRs with higher numbers of uAUG; conversely, TE-high transcripts (red) have shorter 5'UTRs with a much lower number of uAUGs. **$p<0.01$; NS, not significant (one-way ANOVA test).

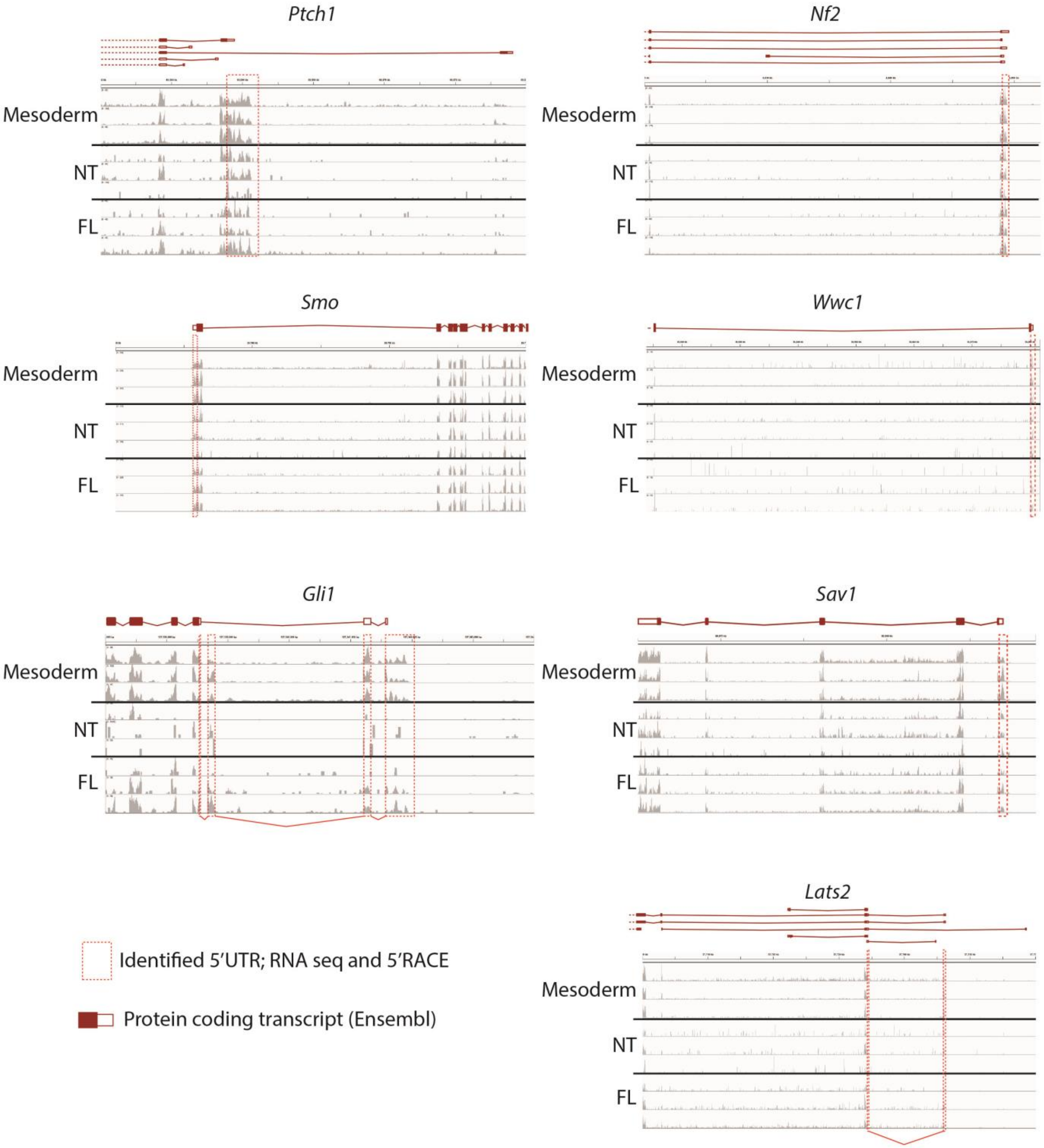

Supplementary Figure 8: Identification of predominant full-length 5'UTRs by 5' RACE and RNA-Seq.
5' RACE on microdissected neural tube and somite was performed to identify the capped full-length 5'UTR of transcripts encoding components of the Shh and Hippo signaling pathways. Longer 5'UTR sequences were obtained by 5' RACE compared to the annotation in Ensembl, for *Ptch1*, *Gli1*, *Wwc1*, and *Sav1*. Alignment of RNA-Seq reads to the 5'UTR region also corroborated the identification of 5'UTR sequences by the 5' RACE experiment as the predominant transcript.

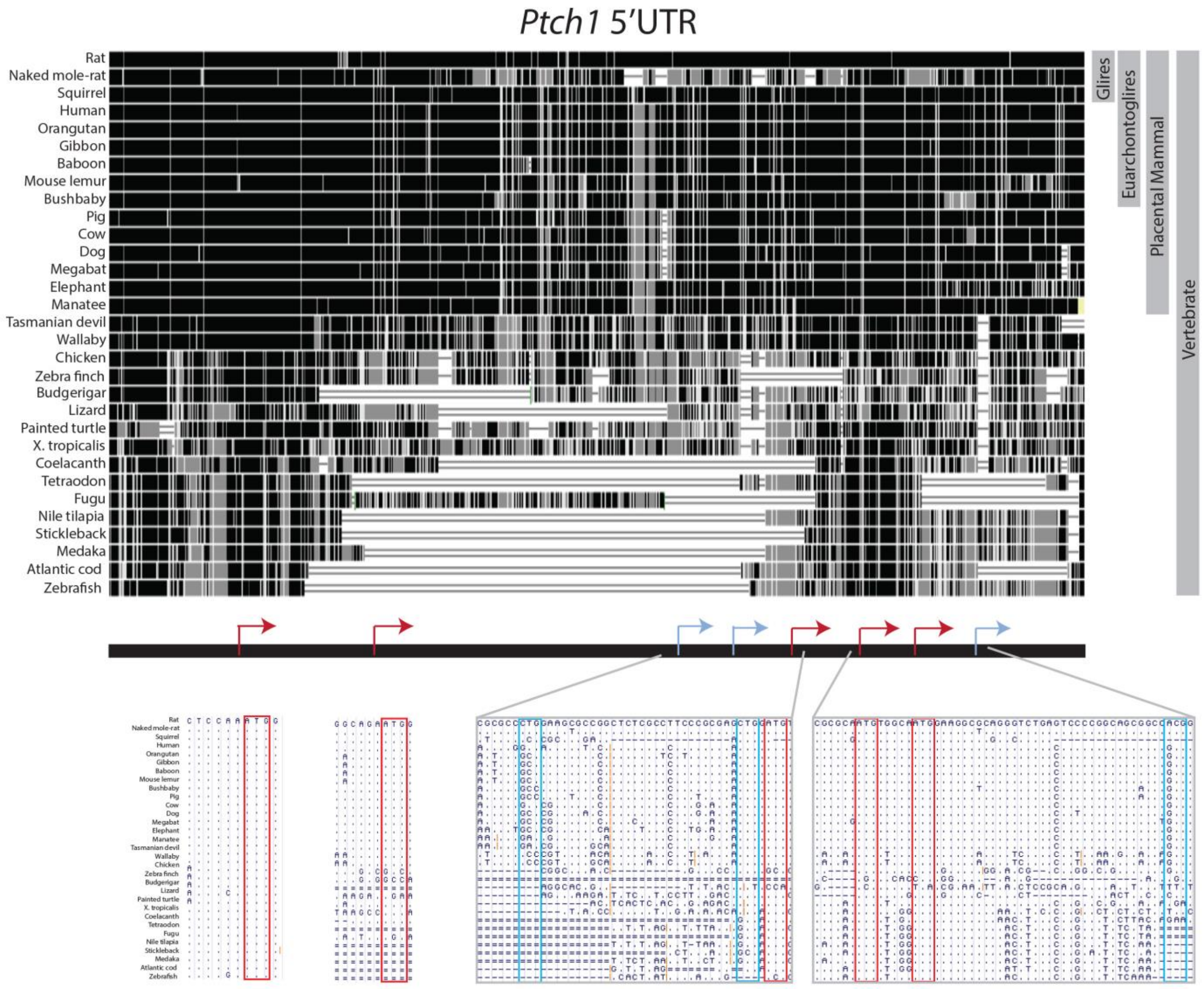

Supplementary Figure 9: Alignment of *Ptch1* 5'UTRs across vertebrates and conservation analysis of uAUG and non-canonical start codons.

(Upper) Alignment of the mouse *Ptch1* 5'UTR (excluding introns) to other vertebrate species from UCSC genome browser. (Lower): A zoom-in view of the sequence alignment from UCSC genome browser, showing the conservation of each uAUG (red boxes) as well as non-canonical (blue boxes) start codons.

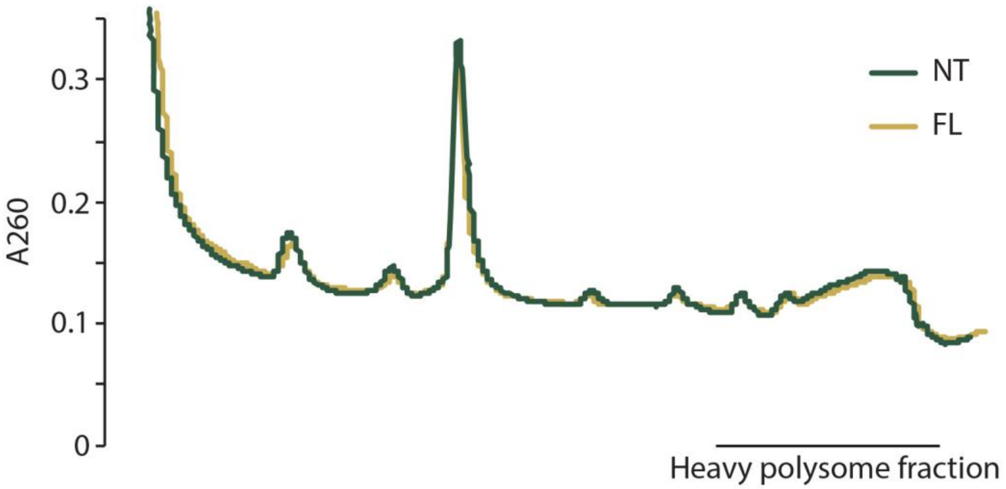

Supplementary Figure 10: Polysome profiles of neural tube and forelimb.
Polysome profiles show no difference in global translation activity between microdissected NT and FL.

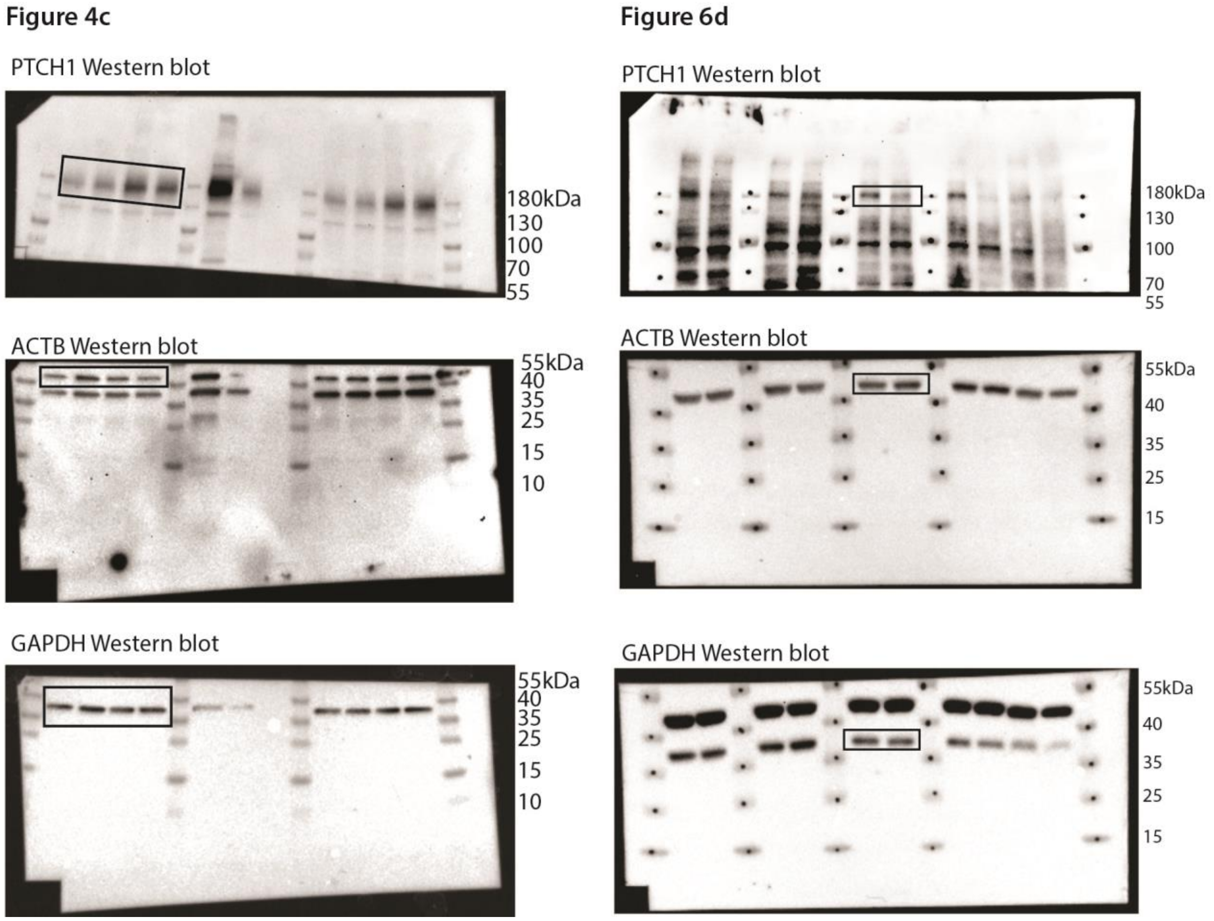

Supplementary Figure 11: Western blot analysis.
Figure shows uncropped Western blots used in Figures 4c and 6d. Black boxes highlight regions of the blot utilized in the indicated Figures.

Supplementary Table 1: 5’ RACE identified 5’UTR sequence for each transcript analyzed in Fig. 3 uAUGs and corresponding stop codons are highlighted in the same color (yellow, green, blue, pink, and grey). uAUGs that initiate an ORF overlapping with the main CDS and corresponding stop codons are shown as blue characters. Non-canonical start codons with favorable Kozak sequences are capitalized. The beginning of main CDS is highlighted in red.

***Ptch1* 5’UTR**

agccaatcgcgttgcagaaacccaagccatctgacagccccgggggcgggagttccagcctttttctctttcgctcgctctttccccctccacccccctcccctcctcctcctctttctccaa
ATGgagagagttcttttttttcttcttccatctcatctattgagtcaaggagctgctgcggccgctgcccgctgcacacacacagagcggagtccccaggtcccggtagcgaaagaag
cgcgcggcgcgggggcagaATGgtccagcgggtcggtgaggagaacaagaaagcagagtccgggaccgagcagccaccgcgaacccagcagccagagagcgaccagcc
gtttcagaagccgccaccgccaactccagccgcagccgcagcagcaccagcagcagcagcggcagccccggcccggcatcgtTGAgactcgccgggtaccgtcgcgtcgcc
cgcagcgggcgccgagggagccggcgcgagcggcgggggtctgcgcgcgccttctgccgTGAaggggtcgcggcgcggggcggggacgtctgggggggacttggggac
cgcgaggcgcgccCTGgaagcgccggctctcgccttcccgcgagCTGgATGtgggcagcggcggccgcaaagacctcgggactcacgcgcaATGtggcaATGga
aggcgcagggtctgagtccccggcagcggccACGgccgcagcacccgcagcgcccgccgtgTGAgcggcagcagcgggtctgtcacccggagccggagtccccggcg
gccagcagcgtcctcgcgagccgagcgcccaggcgcgcccggagcccgcggcggcggcaacATGGCCTCGGCTGGTAACGC

***Smo* 5’UTR**

ccagctagagcaacaaaggagcccggtagtcggcagggagagctcagggggctgcggcgcgctcgcgcggaggtggctgctgggccgcgggCTGgcgtgggggcggagc
cggggacccactcccgcacccccccccccccccccggccggcgcctggcctccatcgaggggCTGggagtcggttttaATGgtgggagagggaATGgggCTGgagat
cggggccccggagggttcccagggtTGAagacagcttcgatctccaggccagggagtccggggtctgtgcatcCTGgcccgggcctgcgctgctcaacATGgggcccgg
gttccaaagtttgcaaagttgggagtcgaggggcccggacgcgcgcggcgcctggcgaaagCTGgccccagactttcggggcgcaccggtcgccTAAgtagcctccgcgg
cccccggggtcgtgtgtgtggccaggggactccggggagctccggggcgcctcagcttttgctgagttggctgtttggccATG

***Gli1* 5’UTR**

atagggtccctcaagggagggggaggatcCTGggggtcCTGggggtgccataagcccggcacccccctctctagcttctatccacccagctcatcttctagaacggtccgaagg
aaggatatacgagggaagtgagcgggaagagccgtagcgtgcgggtggcaacagcgagaaaaagtttttgcaaaggggaaaaaaaaagtttgcgcttctcgcgggtggtcccggc
tcgaggcccggcgggCTGggccggcgggagggCTGgggggccaggttgggagggtggggtggcactgaagctgcgctgcagtggccctgtgacccccctccccgccacac
acctccccccccccccagcccagtttccagccCTGgaccacgcatcccgagcaccgcgccccgacggag>Intron>gtctctttgtccgcgcctctcccacatactagaaatctc
tccctttcttgaggttgggATGaagaagcagttgggacggccagctggaggtctgcgtggtagagggaactcca>Intron>gagactgtggatccccaagactgaacggctgct
tctgcccactctttgggATGtttcttcttaaggaagctgaaaaacgttatTGAtttccATGaccagtttcTGAgATGagggttaga>Intron>ggtcccctcatccttcccT
GAgacgccATG

***Nf2* 5’UTR**

atcttagagaaaaaagtgttagggtgagtcgctgagtagcgtagtgggagtccggagggcggggcggggcggggatccggcaggtcgctgaggtgggggttggtagaggacgct
tcccgagggcgcgcggagcggagCTGgtgacagacacgcacgcgcgtgtgcgctcccaactactgcgcggccccgcgaccctcgggggtcgctgagaggcccggcgtcgg
cgccgcagccgtctaggatttctgtccaaaggcgtccctggcgtccccagcccgaagctccaagctcggggctgcgcgtgcttttgccggcatctccccccgacccccccgtcgtca
gagactgtcccttaactcatctttccgcacccacagccgggtcctcgcggcccATGCTGgccgctggggacccaggcagcctaggtctgtctcgggccggccagccggccac
cATGgtggccctgaggcctgtgcagctactccagggggggcTAAgagacccagaacgcgggcctaggggtgaggggatccaaggccggtacctcgcgccATG

***Wwc1* 5’UTR**

ccgccctcgccgcgCTGggcgcggggaggagacccgggaaccCTGgagcccggcgctccgcctcccgtttccggggcagcgcagttacctgcaccgccggagccacacc
tggcggaggcagaagtctcggggcgcgcggcggggctccggcagaggctgcgggcggcggcggcggcggcggcgtggagcgcagcgcgcagcggcaggggcaggc
aggcggcggctcgggccgctctcctggctggctggccgcggactcgggccggctgggcgccgcggcgggcgcacATGgcagcgTGAgaggcgggcggcgtggggCT
GgggccgcagggccgcATGgacagaggcgccaccccggccggcccccgtTAGggcccccgatccgtgggcgccactccccgatcATGctgcctcggcggccgcctg
gacccaaagcggccagcccgagccgcTGAgccccgtggcggctgcgagctgcATGggggagcgcgggccaggctcgggaagccATGCCCCGGCCGGAG
TTGCCCCTGCCGGAGGGCTGGGAGGAGGCGCGCGACTTCGACGGCAAGGTCTACTACATAGATC

***Sav1* 5’UTR**

ggggcggcccgtgcgcgtcggctttcccagcattcccagcggcgcggggaaagtttaccggacgtaggcggtggccgcagcggtagcggctccgggcggcggaccATGgcc
CTGgtgggcggccgcggccctgtcccaaggcggcctcgggcggcgtcgttgagccggctgacttcccgggaggagctgccctgcgacTGAgcggcggcgggcgcgctta
gcgcggccaacATGcggcggagcctgcgggcgggcgcgggcggcggagacccgaggcgaaggcggcggcggcggcccgggaggcggcggcgggcggcaggctcgc
ggcccTAGcgaggctgctggggcggcggcgggagtcgtccgcgaggATG

***Lats2* 5’UTR**

ccgtggaATGccaacaATGTAGcgaATGtcccgctcgggtctgcgcttcggaaccgcggcgTGAgcgccccgggaagATGgagccgccgccgccagcgccgc
cgccgcttccccgggctcccccgaccctccggggtcagcaaccactgccgccaccggcgcccggtctcccggcgcgcgaggtcccggagccgcgggccaggacgccgccgag
ggtgTAGagcgctcccggag>Intron>agagtgATGgtcttcaaaATGaaaactCTGgaaaattttaggttctctttaggaactacaaaaattgaaggacagcaattttttga
aaggaagttgttcTGAaagcATGtttacaattcacTGAcactgttgactgttctctttaaaataataagacgctttgagaagattgtatttATGgtaaaaggaaaCTGgactaac
aATGAGGCCAAAGACTTTTCCTGCCACAACTTACTCTGGAAATAGCCG

Supplementary Table 2: Plasmid list

Figure	Description	5'UTR	Gene	Marker
Cloning	pGL3-Promoter Vector		Fluc	Amp
Figure. 3b	pRL-SV40 Vector		Rluc	Amp
Figure. 3b	pGL3-HBB	*HBB*	Fluc	Amp
Figure. 3b	pGL3-NF2	*Nf2*	Fluc	Amp
Figure. 3b	pGL3-Wwc1	*Wwc1*	Fluc	Amp
Figure. 3b	pGL3-Sav1	*Sav1*	Fluc	Amp
Figure. 3b	pGL3-Lats2	*Lats2*	Fluc	Amp
Figure. 3b	pGL3-NF2 mut	*Nf2*	Fluc	Amp
Figure. 3b	pGL3-Wwc1 mut	*Wwc1*	Fluc	Amp
Figure. 3b	pGL3-Sav1 mut	*Sav1*	Fluc	Amp
Figure. 3b	pGL3-Lats2 mut	*Lats2*	Fluc	Amp
Figure. 3b	pGL3-Ptch1	*Ptch1*	Fluc	Amp
Figure. 3b	pGL3-Smo	*Smo*	Fluc	Amp
Figure. 3b	pGL3-Gli1	*Gli1*	Fluc	Amp
Figure. 3b	pGL3-Ptch1 mut	*Ptch1*	Fluc	Amp
Figure. 3b	pGL3-Smo mut	*Smo*	Fluc	Amp
Figure. 3b	pGL3-Gli1 mut	*Gli1*	Fluc	Amp
Figure. 3c	pGL3-Gli1 mut for non-canonical	*Gli1*	Fluc	Amp
Figure. 3c	pGL3-Gli1 mut for uAUG	*Gli1*	Fluc	Amp
Figure. 3c	pGL3-Gli1 mut for 2nd exon uAUG	*Gli1*	Fluc	Amp
Figure. 3c	pGL3-Gli1 mut for 3rd exon uAUG	*Gli1*	Fluc	Amp
Figure. 3d	pGL3-Ptch1 mut for non-canonical start site	*Ptch1*	Fluc	Amp
Figure. 3d	pGL3-Ptch1 mut for 1st and 2nd uAUG	*Ptch1*	Fluc	Amp
Figure. 3d	pGL3-Ptch1 mut for 3rd, 4th, and 5th uAUG	*Ptch1*	Fluc	Amp
Figure. 3d	pGL3-Ptch1 deletion 581-705	*Ptch1*	Fluc	Amp
Figure. 3e	pGL3-Ptch1 mut for 4th uAUG	*Ptch1*	Fluc	Amp
Figure. 3f	pGL3-Ptch1 mut for upstream STOP codon	*Ptch1*	Fluc	Amp
Figure. 3f	pGL3-Ptch1 mut for upstream STOP codon +1 frameshift	*Ptch1*	Fluc	Amp
Figure. 3f	pGL3-Ptch1 mut for upstream STOP codon +2 frameshift	*Ptch1*	Fluc	Amp

Supplementary Table 3: qPCR primer list

Gene name	Direction	Sequence
Fluc	Fwd	AAGAGATACGCCCTGGTTC
Fluc	Rev	TTGTATTCAGCCCATATCGTTTC
Rluc	Fwd	TGGAGAATAACTTCTTCGTGGA
Rluc	Rev	TTGGACGACGAACTTCACC
Actb	Fwd	GCCAACCGTGAAAAGATGAC
Actb	Rev	CATCACAATGCCTGTGGTAC
Ptch1	Fwd	ATCATTACACCTTTGGACTGC
Ptch1	Rev	TAAAGGAGGCTTACCTAGGAG
Gli1	Fwd	TCCACCACAAGTCAATAGCTA
Gli1	Rev	CTGGCTGCTCCATAACCCTG
Foxa2	Fwd	ATGCACTCGGCTTCCAGTAT
Foxa2	Rev	TCATTCCAGCGCCCACATAG
Nkx2.2	Fwd	AGGGCCTCCAATACTCCCTG
Nkx2.2	Rev	TCATTGTCCGGTGACTCGTC
Olig2	Fwd	GATGGAGAGATGCGTTCGTT
Olig2	Rev	TGGGGGAAAGAAGTCAAGTG
Pax6	Fwd	ACTTCAGTACCAGGGCAACC
Pax6	Rev	ACTGATGGAGTTGGTGTTCTCTC
Irx3	Fwd	CCGGAGAGTGGAACAGATCG
Irx3	Rev	ACCAGAGCAGCGTCCAGATG
Irx5	Fwd	ACAACTCGCACCTCCAGTAC
Irx5	Rev	TCCCAAGGAACCTGCCATACC
Sox12	Fwd	GTTATTCTAGTGACCGAGACCG
Sox12	Rev	CTAGTTTCTCCCCAGGATTTCC
Ddx5	Fwd	TTCTGATTGCTACCGATGTGG
Ddx5	Rev	GTGTATGCTGTGCCTGTTTTG
Gabbr1	Fwd	ACGAGCTCAAGCTTATCCAC
Gabbr1	Rev	GCATGAGGATGATCTTGATG
Slc4a3	Fwd	CCACATGACCCTGATGCTAAG
Slc4a3	Rev	TCCAAGAAAGGCACGCAG
Arhgef17	Fwd	CGACCTCATGATCAAGCCTG
Arhgef17	Rev	TGCTTGATGTTGCGCTGAGC
Gigyf1	Fwd	TGGGATGACAGAGGCGAGAG
Gigyf1	Rev	TCACTGTCTGAGCGTGCATG
Nsmf	Fwd	TGGAGAAGGAAGAGGACATG
Nsmf	Rev	ACCTTGTAAGGGGTCATGAG
Syngap1	Fwd	ATCAAGTGCACAGCGTCCAG
Syngap1	Rev	GATGCAAACACCTCCTTCAG
Apbb3	Fwd	ACCTAGACAAGTGGAGTTGC
Apbb3	Rev	GTACCAATGGCCTCATTCAG
Kmt2a	Fwd	AGCGTGAGAAGTACTATGAC
Kmt2a	Rev	AAGCGTGCAGCATTTCCATG

Ift172	Fwd	CGCCATGTACCTGGAAGATG
Ift172	Rev	AGTCCTGGTTGTGGACAAAC
Ptch1-b	Fwd	CGGACCGGGACTATCTGCAC
Ptch1-b	Rev	GAGTCTCTGAAACTTCGCTC
Grik5	Fwd	GACATCTTTGAGCTGCAGCG
Grik5	Rev	CACAGATATGGCTCACGGTG
Brd8	Fwd	CAAACTGACTGCTGAGAGAG
Brd8	Rev	CCAGTCTGCTGTCCATGTGC
Gli3	Fwd	CTGATGAAGACCTCCCCAGC
Gli3	Rev	TCCCAGTGGCAGTTTGTCTC
Neur14	Fwd	CTGACCAACGGCAAAGGCAC
Neur14	Rev	GGGCAGAGTTGGACTTCCT
Rai1	Fwd	GAAGCTCTTTGGGCTGCAGG
Rai1	Rev	TAGGAGCACGAGATGGTCGC
Stim2	Fwd	CCTGCACAGAGAAGATAAGCAC
Stim2	Rev	CTATTAACCACTGCAGGGTATCC
Dig4	Fwd	CACAGTGATCTCTTCCAGG
Dig4	Rev	CTGCAACTCATATCCTGGG
Crtc2	Fwd	CCTGTGAAGTCCCTGGAATC
Crtc2	Rev	GGGAAGTGTAGGTTGGTGAG
Uqcrh	Fwd	GAGTACTACTCTGGTTGCGC
Uqcrh	Rev	GGTCCACTAGTTCTTCCTCTTCTTC
Romo1	Fwd	ATTCGGAGTGAGACGTCGAG
Romo1	Rev	ATCTTCACGCGGTCGAAGCAG
Hes1	Fwd	GGAAATGACTGTGAAGCACCTC
Hes1	Rev	AAGCGGGTCACCTCGTTCATG
Tulp3	Fwd	GCGAAGGTTAAAGCCACGAG
Tulp3	Rev	TGGCTTAAGGAAAGCAGCTG
Rbx1	Fwd	GGGCAAGAAGCGCTTTGAAG
Rbx1	Rev	GTTGGCCTGACATTCGATAC
Serp1	Fwd	AACGTCGCTAAGACCTCGAG
Serp1	Rev	TCACATGCCCATCCTGATAC
Ctnnbip1	Fwd	GCATCTGTTTGCCTGAAGTG
Ctnnbip1	Rev	TCTTCCTCAGCATGAGCAGC
Sft2d1	Fwd	TTGCTATGGCTTCCCAATGG
Sft2d1	Rev	CTTCAGTTGCTTCACAGGTC
